# Structure-based assessment of disease-related mutations in human voltage-gated sodium channels

**DOI:** 10.1007/s13238-017-0372-z

**Published:** 2017-02-01

**Authors:** Weiyun Huang, Minhao Liu, S. Frank Yan, Nieng Yan

**Affiliations:** 1 State Key Laboratory of Membrane Biology, School of Life Sciences and School of Medicine, Tsinghua University, Beijing, 100084 China; 2 Beijing Advanced Innovation Center for Structural Biology, School of Life Sciences and School of Medicine, Tsinghua University, Beijing, 100084 China; 3 Tsinghua-Peking Joint Center for Life Sciences, School of Life Sciences and School of Medicine, Tsinghua University, Beijing, 100084 China; 4 Molecular Design and Chemical Biology, Roche Pharma Research and Early Development, Roche Innovation Center Shanghai, Shanghai, 201203 China

**Keywords:** Na_v_ channels, channelopathy, Na_v_1.7, structure modeling, pain

## Abstract

Voltage-gated sodium (Na_v_) channels are essential for the rapid upstroke of action potentials and the propagation of electrical signals in nerves and muscles. Defects of Na_v_ channels are associated with a variety of channelopathies. More than 1000 disease-related mutations have been identified in Na_v_ channels, with Na_v_1.1 and Na_v_1.5 each harboring more than 400 mutations. Na_v_ channels represent major targets for a wide array of neurotoxins and drugs. Atomic structures of Na_v_ channels are required to understand their function and disease mechanisms. The recently determined atomic structure of the rabbit voltage-gated calcium (Ca_v_) channel Ca_v_1.1 provides a template for homology-based structural modeling of the evolutionarily related Na_v_ channels. In this Resource article, we summarized all the reported disease-related mutations in human Na_v_ channels, generated a homologous model of human Na_v_1.7, and structurally mapped disease-associated mutations. Before the determination of structures of human Na_v_ channels, the analysis presented here serves as the base framework for mechanistic investigation of Na_v_ channelopathies and for potential structure-based drug discovery.

**Electronic supplementary material:**

The online version of this article (doi:10.1007/s13238-017-0372-z) contains supplementary material, which is available to authorized users.

## INTRODUCTION

Voltage-gated sodium (Na_v_) channels are essential for the rapid depolarization phase of action potential and play a key role in the electrical signaling in most excitable cells. Structurally, Na_v_ channels are composed of one α subunit and one or more β subunits. The α subunit contains two functionally distinct structural entities, namely, the voltage-sensing domains (VSDs) and the ion-conducting pore domain (Catterall, 2012b, 2014). The β subunits, which bind to α subunit covalently or non-covalently, modulate membrane trafficking, voltage dependence, and channel gating kinetics (Catterall, 2012b, 2014). In mammals, Na_v_ channels have nine known α members distributed in different excitable tissues. Specifically, Na_v_1.1, Na_v_1.2, Na_v_1.3, and Na_v_1.6 are the primary sodium channels in central nervous system (CNS), Na_v_1.4 is primarily expressed in skeletal muscle, Na_v_1.5 is mainly expressed in heart, and Na_v_1.7, Na_v_1.8, and Na_v_1.9 are mainly distributed in peripheral nervous system (Plummer and Meisler, 1999; Goldin, 2001; Catterall et al., 2005).

All α subunits share nearly identical structure topology—a canonical voltage-gated ion channel fold with four homologous repeats, each containing six transmembrane segments S1–S6. Specifically, S5–S6 segments form the pore domain that conducts selective sodium filtering, while S1–S4 segments constitute the voltage-sensing domain that controls voltage-dependent gating (Catterall, 2000). The voltage sensors in the VSDs are featured by a number of positively charged amino acids (arginine or lysine) located at every third position in the S4 segment. Upon membrane depolarization, movements of these charged residues in the S4 segment are coupled to the opening of the pore domain and the subsequent influx of sodium ions across cell membrane. The pore domain is structurally organized with a four-fold pseudo-symmetry. The pore (P) loops, which are supported by the P1 helix (corresponding to the P helix in potassium channel) and P2 helix between S5 and S6 segments in each repeat, constitute the selectivity filter (SF) (Corry and Thomas, 2012). Four amino acid residues (aspartate, glutamate, lysine, and alanine, DEKA, in repeats I, II, III, and IV, respectively) in the P loops are crucial for sodium selectivity. Mutating these residues to glutamates confers calcium selectivity, suggesting that the side chains of these amino acids are likely to interact directly with the sodium ions to determine ion selectivity (Heinemann et al., 1992; Sun et al., 1997).

Na_v_ channels inactivate rapidly. A cluster of hydrophobic amino acids (isoleucine, phenylalanine, methionine, and threonine), namely the IFMT motif, located in the cytosolic regions of domain III and domain IV, are required for rapid inactivation. This is demonstrated by the fact that rapid inactivation could be achieved by titrating small peptides containing the IFMT motif (Vassilev et al., 1988; West et al., 1992).

Sodium channelopathies are a group of diseases caused by defective Na_v_ channels, either, in most cases, of congenital nature or acquired nature (Tables 1, 2, 3, 4, 5, 6, 7, 8, 9, 10) (George, 2005; Catterall, 2012a; Kim, 2014). For example, Na_v_1.1 is primarily expressed in the soma of neuronal cells in the CNS, and mutations of Na_v_1.1 cause GEFS+2 (generalized epilepsy with febrile seizures plus 2) (Catterall et al., 2010). Moreover, mutations of Na_v_1.1 are also the main causes of EIEE6 (epileptic encephalopathy, early infantile, 6) and ICEGTC (intractable childhood epilepsy with generalized tonic-clonic seizures) (Escayg and Goldin, 2010). Na_v_1.5 is the major sodium channel expressed in heart. Na_v_1.5 mutations may lead to various cardiac diseases such as LQT3 (long QT syndrome 3), BRGDA1 (Brugada syndrome 1), and SSS1 (sick sinus syndrome 1) (Olson et al., 2005; Song and Shou, 2012; Veerman et al., 2015). Na_v_1.7 is preferentially expressed in the sympathetic neurons, olfactory epithelium, and dorsal root ganglion sensory neurons, and plays a cardinal role in pain transmission (Djouhri et al., 2003; Dib-Hajj et al., 2013). Gain-of-function mutations of Na_v_1.7 are implicated in two distinct paroxysmal pain syndromes—IEM (primary erythermalgia) and PEPD (paroxysmal extreme pain disorder), while loss-of-function mutations of Na_v_1.7 inflict people with CIP (indifference to pain, congenital, autosomal recessive) (Lampert et al., 2010; Dib-Hajj et al., 2013). In all, Na_v_ channel mutations play a central role in the pathophysiology of sodium channelopathies. Pharmacologic modulation of Na_v_ channels may thereby represent a viable therapeutic approach for the treatment of many neurological disorders such as epilepsy, arrhythmia, and pain.

**Table 1:** Structural mapping of disease-related mutations identified in human Na_v_1.7

Related proteins	Mutations	Diseases	Structural position	Map on hNa_v_1.7
hNa_v_1.7	Q10R	IEM	N-terminus	Q10
hNa_v_1.7	I62V	FEB	N-terminus	I62
hNa_v_1.7	I136V	IEM	DI S1	I136
hNa_v_1.7	P149Q	FEB	DI S1-S2	P149
hNa_v_1.7	R185H	PEPD	DI S3	R185
hNa_v_1.7	R185H	SFN	DI S3	R185
hNa_v_1.7	S211P	IEM	DI S3-S4	S211
hNa_v_1.7	F216S	IEM	DI S4	F216
hNa_v_1.7	I228M	DS	DI S4	I228
hNa_v_1.7	I228M	SFN	DI S4	I228
hNa_v_1.7	I234T	IEM	DI S5	I234
hNa_v_1.7	S241T	IEM	DI S5	S241
hNa_v_1.7	L245V	IEM	DI S5	L245
hNa_v_1.7	N395K	IEM	DI S6	N395
hNa_v_1.7	V400M	IEM	DI S6	V400
hNa_v_1.7	E406K	IEM	DI S6	E406
hNa_v_1.7	S490N	FEB	DI - DII	S490
hNa_v_1.7	E519K	DS	DI - DII	E519
hNa_v_1.7	P610T	IEM	DI - DII	P610
hNa_v_1.7	G616R	IEM	DI - DII	G616
hNa_v_1.7	D623N	SFN	DI - DII	D623
hNa_v_1.7	N641Y	FEB	DI - DII	N641
hNa_v_1.7	K666R	FEB	DI - DII	K666
hNa_v_1.7	K666R	DS	DI - DII	K666
hNa_v_1.7	I695M	DS	DI - DII	I695
hNa_v_1.7	C710Y	DS	DI - DII	C710
hNa_v_1.7	I731K	SFN	DI - DII	I731
hNa_v_1.7	I750V	SFN	DII S1	I750
hNa_v_1.7	I750V	DS	DII S1	I750
hNa_v_1.7	I750V	FEB	DII S1	I750
hNa_v_1.7	L834R	IEM	DII S4	L834
hNa_v_1.7	I859T	IEM	DII S5	I859
hNa_v_1.7	G867D	IEM	DII S5	G867
hNa_v_1.7	L869F	IEM	DII S5	L869
hNa_v_1.7	L869H	IEM	DII S5	L869
hNa_v_1.7	A874P	IEM	DII S5	A874
hNa_v_1.7	V883G	IEM	DII S5	V883
hNa_v_1.7	Q886E	IEM	DII S5	Q886
hNa_v_1.7	R907Q	CIP	DII S5-S6	R907
hNa_v_1.7	M943L	SFN	DII S5-S6	M943
hNa_v_1.7	V1002L	SFN	DII - DIII	V1002
hNa_v_1.7	R1007C	PEPD	DII - DIII	R1007
hNa_v_1.7	L1134F	DS	DII - DIII	L1134
hNa_v_1.7	E1171Q	DS	DII - DIII	E1171
hNa_v_1.7	A1247E	CIP	DIII S2	A1247
hNa_v_1.7	L1278V	DS	DIII S3-S4	L1278
hNa_v_1.7	V1309D	PEPD	DIII S4-S5	V1309
hNa_v_1.7	V1309F	PEPD	DIII S4-S5	V1309
hNa_v_1.7	V1310F	PEPD	DIII S4-S5	V1310
hNa_v_1.7	P1319L	IEM	DIII S4-S5	P1319
hNa_v_1.7	F1460V	IEM	DIII S6	F1460
hNa_v_1.7	I1472T	PEPD	DIII - DIV	I1472
hNa_v_1.7	F1473V	PEPD	DIII - DIV	F1473
hNa_v_1.7	T1475I	PEPD	DIII - DIV	T1475
hNa_v_1.7	M1543I	SFN	DIV S2	M1543
hNa_v_1.7	G1618R	PEPD	DIV S4	G1618
hNa_v_1.7	L1623P	PEPD	DIV S4	L1623
hNa_v_1.7	M1638K	PEPD	DIV S5	M1638
hNa_v_1.7	A1643E	PEPD	DIV S5	A1643
hNa_v_1.7	A1643E	IEM	DIV S5	A1643
hNa_v_1.7	A1643G	IEM	DIV S5	A1643
hNa_v_1.7	A1643T	IEM	DIV S5	A1643
hNa_v_1.7	W1786R	CIP	C-terminus	W1786

IEM: Primary erythermalgia; PEPD: Paroxysmal extreme pain disorder; CIP: Indifference to pain, congenital, autosomal recessive; DS: Dravet syndrome; SFN: Small fiber neuropathy; FEB: Febrile seizures

**Table 2:** Structural mapping of disease-related mutations identified in human Na_v_1.1

Related proteins	Mutations	Diseases	Structural position	Map on hNa_v_1.7
hNa_v_1.1	R27T	GEFS+2	N-terminus	Q25
hNa_v_1.1	S74P	GEFS+2	N-terminus	S72
hNa_v_1.1	D188V	GEFS+2	DI S3	D186
hNa_v_1.1	F218L	GEFS+2	DI S4	F216
hNa_v_1.1	T254I	GEFS+2	DI S5	T252
hNa_v_1.1	S291G	GEFS+2	DI S5-S6	S279
hNa_v_1.1	R377Q	GEFS+2	DI S5-S6	R356
hNa_v_1.1	Y388H	GEFS+2	DI S5-S6	Y367
hNa_v_1.1	Y790C	GEFS+2	DII S1-S2	H766
hNa_v_1.1	R859C	GEFS+2	DII S4	R835
hNa_v_1.1	R859H	GEFS+2	DII S4	R835
hNa_v_1.1	T875M	GEFS+2	DII S4-S5	T851
hNa_v_1.1	I899T	GEFS+2	DII S5	I875
hNa_v_1.1	N935H	GEFS+2	DII S5-S6	N911
hNa_v_1.1	R946H	GEFS+2	DII S5-S6	R922
hNa_v_1.1	M960T	GEFS+2	DII S5-S6	M936
hNa_v_1.1	M973V	GEFS+2	DII S6	M949
hNa_v_1.1	M976I	GEFS+2	DII S6	M952
hNa_v_1.1	I978M	GEFS+2	DII S6	I954
hNa_v_1.1	W1204R	GEFS+2	DII - DIII	W1178
hNa_v_1.1	W1204S	GEFS+2	DII - DIII	W1178
hNa_v_1.1	L1230F	GEFS+2	DIII S1	L1204
hNa_v_1.1	K1249N	GEFS+2	DIII S2	K1223
hNa_v_1.1	T1250M	GEFS+2	DIII S2	I1224
hNa_v_1.1	K1270T	GEFS+2	DIII S2	K1244
hNa_v_1.1	L1309F	GEFS+2	DIII S3-S4	L1283
hNa_v_1.1	V1353L	GEFS+2	DIII S5	V1327
hNa_v_1.1	V1366I	GEFS+2	DIII S5	V1340
hNa_v_1.1	N1414D	GEFS+2	DIII S5-S6	N1388
hNa_v_1.1	V1428A	GEFS+2	DIII S5-S6	V1402
hNa_v_1.1	R1596H	GEFS+2	DIV S2-S3	R1570
hNa_v_1.1	R1648H	GEFS+2	DIV S4	R1622
hNa_v_1.1	I1656M	GEFS+2	DIV S5	I1630
hNa_v_1.1	R1657C	GEFS+2	DIV S5	R1631
hNa_v_1.1	A1685V	GEFS+2	DIV S5	A1659
hNa_v_1.1	F1687S	GEFS+2	DIV S5	F1661
hNa_v_1.1	P1739L	GEFS+2	DIV S5-S6	P1713
hNa_v_1.1	D1742G	GEFS+2	DIV S5-S6	D1716
hNa_v_1.1	F1765L	GEFS+2	DIV S6	Y1739
hNa_v_1.1	E1795K	GEFS+2	C-terminus	E1769
hNa_v_1.1	M1852T	GEFS+2	C-terminus	M1826
hNa_v_1.1	V1857L	GEFS+2	C-terminus	V1831
hNa_v_1.1	D1866Y	GEFS+2	C-terminus	D1840
hNa_v_1.1	I1867T	GEFS+2	C-terminus	I1841
hNa_v_1.1	G58V	EIEE6	N-terminus	G56
hNa_v_1.1	L61F	EIEE6	N-terminus	L59
hNa_v_1.1	F63L	EIEE6	N-terminus	F61
hNa_v_1.1	I68T	EIEE6	N-terminus	I66
hNa_v_1.1	E78D	EIEE6	N-terminus	E76
hNa_v_1.1	D79H	EIEE6	N-terminus	D77
hNa_v_1.1	D79N	EIEE6	N-terminus	D77
hNa_v_1.1	Y84C	EIEE6	N-terminus	Y82
hNa_v_1.1	F90S	EIEE6	N-terminus	F88
hNa_v_1.1	I91T	EIEE6	N-terminus	I89
hNa_v_1.1	A98P	EIEE6	N-terminus	T96
hNa_v_1.1	R101Q	EIEE6	N-terminus	R99
hNa_v_1.1	R101W	EIEE6	N-terminus	R99
hNa_v_1.1	S103G	EIEE6	N-terminus	N101
hNa_v_1.1	T105I	EIEE6	N-terminus	T103
hNa_v_1.1	L108R	EIEE6	N-terminus	L106
hNa_v_1.1	T112I	EIEE6	N-terminus	S110
hNa_v_1.1	R118S	EIEE6	N-terminus	R116
hNa_v_1.1	I124N	EIEE6	N-terminus	I122
hNa_v_1.1	H127D	EIEE6	N-terminus	H125
hNa_v_1.1	T162P	EIEE6	DI S2	T160
hNa_v_1.1	I171K	EIEE6	DI S2	V169
hNa_v_1.1	I171R	EIEE6	DI S2	V169
hNa_v_1.1	A175T	EIEE6	DI S2-23	A173
hNa_v_1.1	A175V	EIEE6	DI S2-S3	A173
hNa_v_1.1	G177E	EIEE6	DI S2-S3	G175
hNa_v_1.1	C179R	EIEE6	DI S2-S3	C177
hNa_v_1.1	W190R	EIEE6	DI S3	W188
hNa_v_1.1	N191K	EIEE6	DI S3	N189
hNa_v_1.1	N191Y	EIEE6	DI S3	N189
hNa_v_1.1	D194G	EIEE6	DI S3	D192
hNa_v_1.1	D194N	EIEE6	DI S3	D192
hNa_v_1.1	T199R	EIEE6	DI S3	V197
hNa_v_1.1	T217K	EIEE6	DI S3-S4	T215
hNa_v_1.1	A223E	EIEE6	DI S4	A221
hNa_v_1.1	T226M	EIEE6	DI S4	T224
hNa_v_1.1	T226R	EIEE6	DI S4	T224
hNa_v_1.1	I227S	EIEE6	DI S4	I225
hNa_v_1.1	I227T	EIEE6	DI S4	I225
hNa_v_1.1	G232S	EIEE6	DI S4-S5	G230
hNa_v_1.1	L233R	EIEE6	DI S5	L231
hNa_v_1.1	A239T	EIEE6	DI S5	A237
hNa_v_1.1	A239V	EIEE6	DI S5	A237
hNa_v_1.1	S243Y	EIEE6	DI S5	S241
hNa_v_1.1	I252N	EIEE6	DI S5	I250
hNa_v_1.1	S259R	EIEE6	DI S5	S257
hNa_v_1.1	G265W	EIEE6	DI S5	G263
hNa_v_1.1	C277R	EIEE6	DI S5-S6	C275
hNa_v_1.1	W280C	EIEE6	DI S5-S6	N278
hNa_v_1.1	W280R	EIEE6	DI S5-S6	N278
hNa_v_1.1	P281A	EIEE6	DI S5-S6	S279
hNa_v_1.1	P281L	EIEE6	DI S5-S6	S279
hNa_v_1.1	P281S	EIEE6	DI S5-S6	S279
hNa_v_1.1	E289V	EIEE6	DI S5-S6	E287
hNa_v_1.1	T297I	EIEE6	DI S5-S6	–
hNa_v_1.1	R322I	EIEE6	DI S5-S6	R301
hNa_v_1.1	S340F	EIEE6	DI S5-S6	T319
hNa_v_1.1	A342V	EIEE6	DI S5-S6	S321
hNa_v_1.1	G343D	EIEE6	DI S5-S6	G322
hNa_v_1.1	C345R	EIEE6	DI S5-S6	C324
hNa_v_1.1	C351W	EIEE6	DI S5-S6	C330
hNa_v_1.1	G355D	EIEE6	DI S5-S6	G334
hNa_v_1.1	R356G	EIEE6	DI S5-S6	R335
hNa_v_1.1	N357I	EIEE6	DI S5-S6	N336
hNa_v_1.1	P358T	EIEE6	DI S5-S6	P357
hNa_v_1.1	N359S	EIEE6	DI S5-S6	D338
hNa_v_1.1	T363P	EIEE6	DI S5-S6	T342
hNa_v_1.1	T363R	EIEE6	DI S5-S6	T342
hNa_v_1.1	D366E	EIEE6	DI S5-S6	D345
hNa_v_1.1	L378Q	EIEE6	DI S5-S6	L357
hNa_v_1.1	M379R	EIEE6	DI S5-S6	M358
hNa_v_1.1	F383L	EIEE6	DI S5-S6	Y362
hNa_v_1.1	W384R	EIEE6	DI S5-S6	M363
hNa_v_1.1	R393C	EIEE6	DI S5-S6	R372
hNa_v_1.1	R393H	EIEE6	DI S5-S6	R372
hNa_v_1.1	R393S	EIEE6	DI S5-S6	R372
hNa_v_1.1	M400V	EIEE6	DI S5-S6	M379
hNa_v_1.1	F403L	EIEE6	DI S6	F383
hNa_v_1.1	F403V	EIEE6	DI S6	F382
hNa_v_1.1	V406F	EIEE6	DI S6	V385
hNa_v_1.1	L409W	EIEE6	DI S6	L388
hNa_v_1.1	Y413N	EIEE6	DI S6	Y392
hNa_v_1.1	Y426C	EIEE6	DI S6	Y405
hNa_v_1.1	Y426N	EIEE6	DI S6	Y405
hNa_v_1.1	S525F	EIEE6	DI - DII	S505
hNa_v_1.1	S626G	EIEE6	DI - DII	S606
hNa_v_1.1	D674G	EIEE6	DI - DII	D651
hNa_v_1.1	N762D	EIEE6	DI - DII	Y738
hNa_v_1.1	L783P	EIEE6	DII S1	L759
hNa_v_1.1	M785T	EIEE6	DII S1-S2	M761
hNa_v_1.1	T812I	EIEE6	DII S2	A788
hNa_v_1.1	T812R	EIEE6	DII S2	A788
hNa_v_1.1	L842R	EIEE6	DII S3	L818
hNa_v_1.1	S843R	EIEE6	DII S3	S819
hNa_v_1.1	E846K	EIEE6	DII S3	E822
hNa_v_1.1	R859C	EIEE6	DII S4	R835
hNa_v_1.1	R862Q	EIEE6	DII S4	R838
hNa_v_1.1	R865G	EIEE6	DII S4	R841
hNa_v_1.1	T875K	EIEE6	DII S4-S5	T851
hNa_v_1.1	T875M	EIEE6	DII S4-S5	T851
hNa_v_1.1	L876I	EIEE6	DII S5	L852
hNa_v_1.1	L890P	EIEE6	DII S5	L866
hNa_v_1.1	V896F	EIEE6	DII S5	V872
hNa_v_1.1	V896L	EIEE6	DII S5	V872
hNa_v_1.1	F902C	EIEE6	DII S5	F878
hNa_v_1.1	C927F	EIEE6	DII S5-S6	C903
hNa_v_1.1	R931C	EIEE6	DII S5-S6	R907
hNa_v_1.1	W932C	EIEE6	DII S5-S6	W908
hNa_v_1.1	H933P	EIEE6	DII S5-S6	H909
hNa_v_1.1	M934I	EIEE6	DII S5-S6	M910
hNa_v_1.1	H939P	EIEE6	DII S5-S6	H915
hNa_v_1.1	H939Q	EIEE6	DII S5-S6	H915
hNa_v_1.1	H939Y	EIEE6	DII S5-S6	H915
hNa_v_1.1	S940F	EIEE6	DII S5-S6	S916
hNa_v_1.1	L942P	EIEE6	DII S5-S6	L918
hNa_v_1.1	I943N	EIEE6	DII S5-S6	I919
hNa_v_1.1	V944A	EIEE6	DII S5-S6	V920
hNa_v_1.1	V944E	EIEE6	DII S5-S6	V920
hNa_v_1.1	F945L	EIEE6	DII S5-S6	F921
hNa_v_1.1	R946C	EIEE6	DII S5-S6	R922
hNa_v_1.1	R946H	EIEE6	DII S5-S6	R922
hNa_v_1.1	R946S	EIEE6	DII S5-S6	R922
hNa_v_1.1	C949S	EIEE6	DII S5-S6	C925
hNa_v_1.1	C949Y	EIEE6	DII S5-S6	C925
hNa_v_1.1	G950E	EIEE6	DII S5-S6	G926
hNa_v_1.1	G950R	EIEE6	DII S5-S6	G926
hNa_v_1.1	W952G	EIEE6	DII S5-S6	W928
hNa_v_1.1	E954K	EIEE6	DII S5-S6	E930
hNa_v_1.1	M956K	EIEE6	DII S5-S6	M932
hNa_v_1.1	W957L	EIEE6	DII S5-S6	W933
hNa_v_1.1	C959R	EIEE6	DII S5-S6	C935
hNa_v_1.1	M960V	EIEE6	DII S5-S6	M936
hNa_v_1.1	M973K	EIEE6	DII S6	M949
hNa_v_1.1	M976I	EIEE6	DII S6	M952
hNa_v_1.1	G979V	EIEE6	DII S6	G955
hNa_v_1.1	N985I	EIEE6	DII S6	N961
hNa_v_1.1	L986F	EIEE6	DII S6	L962
hNa_v_1.1	L986P	EIEE6	DII S6	L962
hNa_v_1.1	F987L	EIEE6	DII S6	F963
hNa_v_1.1	S993R	EIEE6	DII - DIII	S969
hNa_v_1.1	D998G	EIEE6	DII - DIII	D974
hNa_v_1.1	E1068K	EIEE6	DII - DIII	E1045
hNa_v_1.1	L1207P	EIEE6	DII - DIII	I1181
hNa_v_1.1	R1208K	EIEE6	DII - DIII	R1182
hNa_v_1.1	T1210K	EIEE6	DII - DIII	T1184
hNa_v_1.1	E1221K	EIEE6	DIII S1	E1195
hNa_v_1.1	L1230F	EIEE6	DIII S1	L1204
hNa_v_1.1	S1231R	EIEE6	DIII S1	S1205
hNa_v_1.1	S1231T	EIEE6	DIII S1	S1205
hNa_v_1.1	G1233R	EIEE6	DIII S1	G1207
hNa_v_1.1	E1238D	EIEE6	DIII S1-S2	E1212
hNa_v_1.1	D1239G	EIEE6	DIII S1-S2	D1213
hNa_v_1.1	D1239Y	EIEE6	DIII S1-S2	D1213
hNa_v_1.1	R1245Q	EIEE6	DIII S1-S2	K1219
hNa_v_1.1	A1255D	EIEE6	DIII S2	A1229
hNa_v_1.1	T1260P	EIEE6	DIII S2	T1234
hNa_v_1.1	F1263L	EIEE6	DIII S2	F1237
hNa_v_1.1	L1265P	EIEE6	DIII S2	L1239
hNa_v_1.1	E1266A	EIEE6	DIII S2	E1240
hNa_v_1.1	G1275V	EIEE6	DIII S2-S3	G1249
hNa_v_1.1	W1284S	EIEE6	DIII S3	W1258
hNa_v_1.1	L1287P	EIEE6	DIII S3	L1261
hNa_v_1.1	D1288N	EIEE6	DIII S3	D1262
hNa_v_1.1	R1316G	EIEE6	DIII S4	R1290
hNa_v_1.1	R1316S	EIEE6	DIII S4	R1290
hNa_v_1.1	A1320V	EIEE6	DIII S4	A1294
hNa_v_1.1	A1326P	EIEE6	DIII S4	A1300
hNa_v_1.1	S1328P	EIEE6	DIII S4-S5	S1302
hNa_v_1.1	V1335M	EIEE6	DIII S4-S5	V1309
hNa_v_1.1	A1339V	EIEE6	DIII S4-S5	A1313
hNa_v_1.1	I1344M	EIEE6	DIII S4-S5	I1318
hNa_v_1.1	V1350G	EIEE6	DIII S5	V1324
hNa_v_1.1	L1355P	EIEE6	DIII S5	L1329
hNa_v_1.1	W1358R	EIEE6	DIII S5	W1332
hNa_v_1.1	W1358S	EIEE6	DIII S5	W1332
hNa_v_1.1	N1367K	EIEE6	DIII S5	N1341
hNa_v_1.1	A1370P	EIEE6	DIII S5-S6	A1344
hNa_v_1.1	N1378H	EIEE6	DIII S5-S6	N1352
hNa_v_1.1	N1378T	EIEE6	DIII S5-S6	N1352
hNa_v_1.1	F1385V	EIEE6	DIII S5-S6	F1359
hNa_v_1.1	V1390M	EIEE6	DIII S5-S6	V1364
hNa_v_1.1	N1391S	EIEE6	DIII S5-S6	P1365
hNa_v_1.1	H1393P	EIEE6	DIII S5-S6	R1367
hNa_v_1.1	T1394I	EIEE6	DIII S5-S6	S1368
hNa_v_1.1	C1396G	EIEE6	DIII S5-S6	C1370
hNa_v_1.1	C1396Y	EIEE6	DIII S5-S6	C1370
hNa_v_1.1	N1414Y	EIEE6	DIII S5-S6	N1388
hNa_v_1.1	D1416G	EIEE6	DIII S5-S6	D1390
hNa_v_1.1	N1417S	EIEE6	DIII S5-S6	N1391
hNa_v_1.1	V1418G	EIEE6	DIII S5-S6	V1392
hNa_v_1.1	Y1422C	EIEE6	DIII S5-S6	Y1396
hNa_v_1.1	L1423F	EIEE6	DIII S5-S6	L1397
hNa_v_1.1	L1426R	EIEE6	DIII S5-S6	L1400
hNa_v_1.1	Q1427P	EIEE6	DIII S5-S6	Q1401
hNa_v_1.1	F1431I	EIEE6	DIII S5-S6	F1405
hNa_v_1.1	G1433E	EIEE6	DIII S5-S6	G1407
hNa_v_1.1	G1433R	EIEE6	DIII S5-S6	G1407
hNa_v_1.1	G1433V	EIEE6	DIII S5-S6	G1407
hNa_v_1.1	W1434R	EIEE6	DIII S5-S6	W1408
hNa_v_1.1	I1437M	EIEE6	DIII S5-S6	I1411
hNa_v_1.1	A1441P	EIEE6	DIII S5-S6	A1415
hNa_v_1.1	Q1450K	EIEE6	DIII S5-S6	Q1424
hNa_v_1.1	Q1450R	EIEE6	DIII S5-S6	Q1424
hNa_v_1.1	P1451L	EIEE6	DIII S5-S6	P1425
hNa_v_1.1	P1451S	EIEE6	DIII S5-S6	P1425
hNa_v_1.1	Y1453C	EIEE6	DIII S5-S6	Y1427
hNa_v_1.1	E1454K	EIEE6	DIII S5-S6	E1428
hNa_v_1.1	L1461I	EIEE6	DIII S6	I1435
hNa_v_1.1	Y1462C	EIEE6	DIII S6	Y1436
hNa_v_1.1	Y1462H	EIEE6	DIII S6	Y1436
hNa_v_1.1	F1463S	EIEE6	DIII S6	F1437
hNa_v_1.1	G1470W	EIEE6	DIII S6	G1444
hNa_v_1.1	F1472S	EIEE6	DIII S6	F1446
hNa_v_1.1	L1475S	EIEE6	DIII S6	L1449
hNa_v_1.1	N1476K	EIEE6	DIII S6	N1450
hNa_v_1.1	D1484G	EIEE6	DIII S6	D1458
hNa_v_1.1	N1485Y	EIEE6	DIII S6	N1459
hNa_v_1.1	E1503K	EIEE6	DIII - DIV	E1477
hNa_v_1.1	L1514S	EIEE6	DIII - DIV	L1488
hNa_v_1.1	V1538I	EIEE6	DIII - DIV	V1512
hNa_v_1.1	D1544A	EIEE6	DIV S1	D1518
hNa_v_1.1	D1544G	EIEE6	DIV S1	D1518
hNa_v_1.1	I1545V	EIEE6	DIV S1	I1519
hNa_v_1.1	M1555R	EIEE6	DIV S1	M1529
hNa_v_1.1	E1561K	EIEE6	DIV S1-S2	E1535
hNa_v_1.1	V1579E	EIEE6	DIV S2	V1553
hNa_v_1.1	G1586E	EIEE6	DIV S2	G1560
hNa_v_1.1	C1588R	EIEE6	DIV S2	C1562
hNa_v_1.1	L1592H	EIEE6	DIV S2	L1566
hNa_v_1.1	L1592P	EIEE6	DIV S2	L1566
hNa_v_1.1	R1596C	EIEE6	DIV S2-S3	R1570
hNa_v_1.1	R1596L	EIEE6	DIV S2-S3	R1570
hNa_v_1.1	N1605S	EIEE6	DIV S3	N1579
hNa_v_1.1	D1608G	EIEE6	DIV S3	D1582
hNa_v_1.1	D1608Y	EIEE6	DIV S3	D1582
hNa_v_1.1	V1612I	EIEE6	DIV S3	V1586
hNa_v_1.1	V1630L	EIEE6	DIV S3-S4	V1604
hNa_v_1.1	V1630M	EIEE6	DIV S3-S4	V1604
hNa_v_1.1	V1637E	EIEE6	DIV S4	V1611
hNa_v_1.1	I1638N	EIEE6	DIV S4	I1612
hNa_v_1.1	I1638T	EIEE6	DIV S4	I1612
hNa_v_1.1	R1639G	EIEE6	DIV S4	R1613
hNa_v_1.1	R1642S	EIEE6	DIV S4	R1616
hNa_v_1.1	R1645Q	EIEE6	DIV S4	R1619
hNa_v_1.1	R1648C	EIEE6	DIV S4	R1622
hNa_v_1.1	R1648H	EIEE6	DIV S4	R1622
hNa_v_1.1	A1653E	EIEE6	DIV S4-S5	A1627
hNa_v_1.1	T1658M	EIEE6	DIV S5	T1632
hNa_v_1.1	T1658R	EIEE6	DIV S5	T1632
hNa_v_1.1	L1660P	EIEE6	DIV S5	L1634
hNa_v_1.1	F1661S	EIEE6	DIV S5	F1635
hNa_v_1.1	A1662V	EIEE6	DIV S5	A1636
hNa_v_1.1	M1664K	EIEE6	DIV S5	M1638
hNa_v_1.1	L1667P	EIEE6	DIV S5	L1641
hNa_v_1.1	P1668A	EIEE6	DIV S5	P1642
hNa_v_1.1	P1668L	EIEE6	DIV S5	P1642
hNa_v_1.1	N1672I	EIEE6	DIV S5	N1646
hNa_v_1.1	I1673T	EIEE6	DIV S5	I1647
hNa_v_1.1	G1674R	EIEE6	DIV S5	G1648
hNa_v_1.1	L1675R	EIEE6	DIV S5	L1649
hNa_v_1.1	L1677F	EIEE6	DIV S5	L1651
hNa_v_1.1	I1683T	EIEE6	DIV S5	I1657
hNa_v_1.1	Y1684D	EIEE6	DIV S5	Y1658
hNa_v_1.1	A1685D	EIEE6	DIV S5	A1659
hNa_v_1.1	G1688W	EIEE6	DIV S5	G1662
hNa_v_1.1	F1692S	EIEE6	DIV S5	F1666
hNa_v_1.1	Y1694C	EIEE6	DIV S5-S6	Y1668
hNa_v_1.1	F1707V	EIEE6	DIV S5-S6	F1681
hNa_v_1.1	S1713N	EIEE6	DIV S5-S6	S1687
hNa_v_1.1	M1714K	EIEE6	DIV S5-S6	M1688
hNa_v_1.1	M1714R	EIEE6	DIV S5-S6	M1688
hNa_v_1.1	C1716R	EIEE6	DIV S5-S6	C1690
hNa_v_1.1	T1721R	EIEE6	DIV S5-S6	T1695
hNa_v_1.1	G1725C	EIEE6	DIV S5-S6	G1699
hNa_v_1.1	W1726R	EIEE6	DIV S5-S6	W1700
hNa_v_1.1	D1727G	EIEE6	DIV S5-S6	D1701
hNa_v_1.1	C1741R	EIEE6	DIV S5-S6	C1715
hNa_v_1.1	G1749E	EIEE6	DIV S5-S6	G1723
hNa_v_1.1	C1756G	EIEE6	DIV S5-S6	C1730
hNa_v_1.1	G1762E	EIEE6	DIV S6	G1736
hNa_v_1.1	I1763N	EIEE6	DIV S6	I1737
hNa_v_1.1	I1770F	EIEE6	DIV S6	I1744
hNa_v_1.1	I1770N	EIEE6	DIV S6	I1744
hNa_v_1.1	I1770T	EIEE6	DIV S6	I1744
hNa_v_1.1	I1771F	EIEE6	DIV S6	I1745
hNa_v_1.1	I1771N	EIEE6	DIV S6	I1745
hNa_v_1.1	S1773F	EIEE6	DIV S6	S1747
hNa_v_1.1	M1780T	EIEE6	DIV S6	M1754
hNa_v_1.1	Y1781C	EIEE6	DIV S6	Y1755
hNa_v_1.1	Y1781H	EIEE6	DIV S6	Y1755
hNa_v_1.1	I1782M	EIEE6	DIV S6	I1756
hNa_v_1.1	I1782S	EIEE6	DIV S6	I1756
hNa_v_1.1	A1783T	EIEE6	DIV S6	A1757
hNa_v_1.1	A1783V	EIEE6	DIV S6	A1757
hNa_v_1.1	E1787K	EIEE6	DIV S6	E1761
hNa_v_1.1	N1788K	EIEE6	DIV S6	N1862
hNa_v_1.1	A1792T	EIEE6	C-terminus	A1766
hNa_v_1.1	F1808I	EIEE6	C-terminus	F1782
hNa_v_1.1	W1812G	EIEE6	C-terminus	W1786
hNa_v_1.1	W1812S	EIEE6	C-terminus	W1786
hNa_v_1.1	F1831S	EIEE6	C-terminus	F1805
hNa_v_1.1	A1832P	EIEE6	C-terminus	A1806
hNa_v_1.1	L1835F	EIEE6	C-terminus	L1809
hNa_v_1.1	M1852K	EIEE6	C-terminus	M1826
hNa_v_1.1	P1855L	EIEE6	C-terminus	P1829
hNa_v_1.1	G1880E	EIEE6	C-terminus	G1854
hNa_v_1.1	E1881D	EIEE6	C-terminus	E1855
hNa_v_1.1	T1909I	EIEE6	C-terminus	T1883
hNa_v_1.1	I1922T	EIEE6	C-terminus	I1896
hNa_v_1.1	F90S	ICEGTC	N-terminus	F88
hNa_v_1.1	R101Q	ICEGTC	N-terminus	R99
hNa_v_1.1	F178S	ICEGTC	DI S2-S3	F176
hNa_v_1.1	I252M	ICEGTC	DI S5	I250
hNa_v_1.1	H290R	ICEGTC	DI S5-S6	S288
hNa_v_1.1	R393H	ICEGTC	DI S5-S6	R372
hNa_v_1.1	T808S	ICEGTC	DII S2	T784
hNa_v_1.1	V896I	ICEGTC	DII S5	V872
hNa_v_1.1	V944A	ICEGTC	DII S5-S6	R920
hNa_v_1.1	G979R	ICEGTC	DII S6	G955
hNa_v_1.1	V983A	ICEGTC	DII S6	V959
hNa_v_1.1	N1011I	ICEGTC	DII - DIII	N987
hNa_v_1.1	R1213Q	ICEGTC	DII - DIII	K1187
hNa_v_1.1	Y1254C	ICEGTC	DIII S2	Y1228
hNa_v_1.1	R1325T	ICEGTC	DIII S4	R1299
hNa_v_1.1	S1328P	ICEGTC	DIII S4-S5	S1302
hNa_v_1.1	F1357L	ICEGTC	DIII S5	F1331
hNa_v_1.1	V1366I	ICEGTC	DIII S5	V1340
hNa_v_1.1	C1376R	ICEGTC	DIII S5-S6	C1350
hNa_v_1.1	A1429D	ICEGTC	DIII S5-S6	A1403
hNa_v_1.1	Y1462H	ICEGTC	DIII S6	Y1436
hNa_v_1.1	M1511K	ICEGTC	DIII - DIV	M1485
hNa_v_1.1	V1611F	ICEGTC	DIV S3	V1585
hNa_v_1.1	M1619V	ICEGTC	DIV S3	M1593
hNa_v_1.1	P1632S	ICEGTC	DIV S3-S4	P1606
hNa_v_1.1	Y1684S	ICEGTC	DIV S5	Y1658
hNa_v_1.1	T1709I	ICEGTC	DIV S5-S6	T1683
hNa_v_1.1	A1724P	ICEGTC	DIV S5-S6	A1698
hNa_v_1.1	Y1781C	ICEGTC	DIV S6	Y1755
hNa_v_1.1	F1808L	ICEGTC	C-terminus	F1782
hNa_v_1.1	R1861W	ICEGTC	C-terminus	R1835
hNa_v_1.1	T1174S	FHM3	DII - DIII	S1148
hNa_v_1.1	Q1489H	FHM3	DIII S6	Q1463
hNa_v_1.1	Q1489K	FHM3	DIII S6	Q1463
hNa_v_1.1	F1499L	FHM3	DIII - DIV	F1473
hNa_v_1.1	L1649Q	FHM3	DIV S4	L1623
hNa_v_1.1	M145T	FEB3A	DI S1	M143
hNa_v_1.1	E1308D	FEB3A	DIII S3-S4	D1282

GEFS+2: Generalized epilepsy with febrile seizures plus 2; EIEE6: Epileptic encephalopathy, early infantile, 6; ICEGTC: Intractable childhood epilepsy with generalized tonic-clonic seizures; FHM3: Migraine, familial hemiplegic, 3; FEB3A: Febrile seizures, familial, 3A

**Table 3:** Structural mapping of disease-related mutations identified in human Na_v_1.2

Related proteins	Mutations	Diseases	Structural position	Map on hNa_v_1.7
hNa_v_1.2	E169G	EIEE11	DI S2	E166
hNa_v_1.2	R188W	BFIS3	DI S3	R185
hNa_v_1.2	V208E	BFIS3	DI S3-S4	V205
hNa_v_1.2	N212D	EIEE11	DI S3-S4	N209
hNa_v_1.2	V213D	EIEE11	DI S3-S4	V210
hNa_v_1.2	R223Q	BFIS3	DI S4	R220
hNa_v_1.2	T236S	EIEE11	DI S5	T233
hNa_v_1.2	M252V	BFIS3	DI S5	M249
hNa_v_1.2	V261M	BFIS3	DI S5	V258
hNa_v_1.2	A263T	EIEE11	DI S5	A260
hNa_v_1.2	A263V	EIEE11	DI S5	A260
hNa_v_1.2	D322N	DS	DI - DII	D298
hNa_v_1.2	F328V	DS	DI - DII	Y305
hNa_v_1.2	E430Q	BFIS3	DI - DII	E407
hNa_v_1.2	D649N	DS	DI - DII	D623
hNa_v_1.2	R853Q	EIEE11	DII S4	R838
hNa_v_1.2	N876T	EIEE11	DII S5	N861
hNa_v_1.2	V892I	BFIS3	DII S5	V877
hNa_v_1.2	E999K	EIEE11	DII - DIII	D984
hNa_v_1.2	N1001K	BFIS3	DII - DIII	N986
hNa_v_1.2	L1003I	BFIS3	DII - DIII	L988
hNa_v_1.2	E1211K	EIEE11	DIII S1	E1195
hNa_v_1.2	R1312T	EIEE11	DIII S4	R1296
hNa_v_1.2	R1312T	DS	DIII S4	R1296
hNa_v_1.2	R1319Q	BFIS3	DIII S4-S5	R1303
hNa_v_1.2	M1323V	EIEE11	DIII S4-S5	M1307
hNa_v_1.2	V1326L	EIEE11	DIII S4-S5	V1310
hNa_v_1.2	V1326D	EIEE11	DIII S4-S5	V1310
hNa_v_1.2	L1330F	BFIS3	DIII S4-S5	L1314
hNa_v_1.2	S1336Y	EIEE11	DIII S4-S5	S1320
hNa_v_1.2	M1338T	EIEE11	DIII S5	M1322
hNa_v_1.2	L1342P	BFIS3	DIII S5	L1326
hNa_v_1.2	I1473M	EIEE11	DIII S6	I1457
hNa_v_1.2	L1563V	BFIS3	DIV S2	L1547
hNa_v_1.2	Y1589C	BFIS3	DIV S2-S3	Y1573
hNa_v_1.2	I1596S	BFIS3	DIV S3	I1580
hNa_v_1.2	T1623N	EIEE11	DIV S3-S4	T1607
hNa_v_1.2	R1629L	EIEE11	DIV S4	R1613
hNa_v_1.2	L1660Y	EIEE11	DIV S5	L1644
hNa_v_1.2	R1918H	BFIS3	C-terminus	R1902

BFIS3: Seizures, benign familial infantile 3; EIEE11: Epileptic encephalopathy, early infantile, 11; DS: Dravet syndrome

**Table 4:** Structural mapping of disease-related mutations identified in human Na_v_1.3

Related proteins	Mutations	Diseases	Structural position	Map on hNa_v_1.7
hNa_v_1.3	K354Q	CPE	DI - DII	K332
hNa_v_1.3	R357Q	CPE	DI - DII	R335
hNa_v_1.3	D815N	CPE	DII S2-S3	D799
hNa_v_1.3	E1160K	CPE	DII - DIII	M1146
hNa_v_1.3	M1372V	CPE	DIII S5-S6	R1358
hNa_v_1.3	G1862C	CPE	C-terminus	G1851

CPE: Cryptogenic partial epilepsy

**Table 5:** Structural mapping of disease-related mutations identified in human Na_v_1.4

Related proteins	Mutations	Diseases	Structural position	Map on hNa_v_1.7
hNa_v_1.4	Q270K	PMC	DI S5	Q265
hNa_v_1.4	I693T	PMC	DII S5	I858
hNa_v_1.4	T704M	PMC	DII S5	T870
hNa_v_1.4	S804F	PMC	DII - DIII	S970
hNa_v_1.4	A1152D	PMC	DIII S4-S5	A1313
hNa_v_1.4	A1156T	PMC	DIII S4-S5	A1317
hNa_v_1.4	V1293I	PMC	DIII S6	V1455
hNa_v_1.4	G1306A	PMC	DIII S6	G1468
hNa_v_1.4	G1306E	PMC	DIII S6	G1468
hNa_v_1.4	G1306V	PMC	DIII S6	G1468
hNa_v_1.4	T1313M	PMC	DIII - DIV	T1475
hNa_v_1.4	L1433R	PMC	DIV S3	L1595
hNa_v_1.4	L1436P	PMC	DIV S3	L1598
hNa_v_1.4	R1448C	PMC	DIV S4	R1610
hNa_v_1.4	R1448H	PMC	DIV S4	R1610
hNa_v_1.4	R1448L	PMC	DIV S4	R1610
hNa_v_1.4	G1456E	PMC	DIV S4	G1618
hNa_v_1.4	F1473S	PMC	DIV S5	F1635
hNa_v_1.4	V1589M	PMC	DIV S6	V1751
hNa_v_1.4	F1705I	PMC	C-terminus	F1867
hNa_v_1.4	R222W	HOKPP2	DI S4	E217
hNa_v_1.4	R669H	HOKPP2	DII S4	R835
hNa_v_1.4	R672C	HOKPP2	DII S4	R838
hNa_v_1.4	R672G	HOKPP2	DII S4	R838
hNa_v_1.4	R672H	HOKPP2	DII S4	R838
hNa_v_1.4	R672S	HOKPP2	DII S4	R838
hNa_v_1.4	R1129Q	HOKPP2	DIII S4	R1290
hNa_v_1.4	R1132Q	HOKPP2	DIII S4	R1293
hNa_v_1.4	R1135C	HOKPP2	DIII S4	R1296
hNa_v_1.4	R1135H	HOKPP2	DIII S4	R1299
hNa_v_1.4	P1158S	HOKPP2	DIII S4-S5	P1319
hNa_v_1.4	T704M	HYPP	DII S5	T870
hNa_v_1.4	V781I	HYPP	DII S6	V947
hNa_v_1.4	A1156T	HYPP	DIII S4-S5	A1317
hNa_v_1.4	L1433R	HYPP	DIV S3	L1595
hNa_v_1.4	M1592V	HYPP	DIV S6	M1754
hNa_v_1.4	R675G	NKPP	DII S4	R841
hNa_v_1.4	R675Q	NKPP	DII S4	R841
hNa_v_1.4	R675W	NKPP	DII S4	R841
hNa_v_1.4	V781I	NKPP	DII S6	V947
hNa_v_1.4	R1129Q	NKPP	DIII S4	R1290
hNa_v_1.4	M1592V	NKPP	DIV S6	M1754
hNa_v_1.4	I141V	MYOSCN4A	DI S1	I136
hNa_v_1.4	R225W	MYOSCN4A	DI S4	R220
hNa_v_1.4	N440K	MYOSCN4A	DI S6	N395
hNa_v_1.4	V445M	MYOSCN4A	DI - DII	V440
hNa_v_1.4	E452K	MYOSCN4A	DI - DII	E447
hNa_v_1.4	I588V	MYOSCN4A	DII S1	I754
hNa_v_1.4	F671S	MYOSCN4A	DII S4	F837
hNa_v_1.4	A715T	MYOSCN4A	DII S5	A881
hNa_v_1.4	S804N	MYOSCN4A	DII - DIII	S970
hNa_v_1.4	A1156T	MYOSCN4A	DIII S4-S5	A1317
hNa_v_1.4	P1158L	MYOSCN4A	DIII S4-S5	P1319
hNa_v_1.4	I1160V	MYOSCN4A	DIII S4-S5	I1321
hNa_v_1.4	N1297K	MYOSCN4A	DIII S6	I1457
hNa_v_1.4	G1306E	MYOSCN4A	DIII S6	G1468
hNa_v_1.4	G1306V	MYOSCN4A	DIII S6	G1468
hNa_v_1.4	I1310N	MYOSCN4A	DIII - DIV	I1472
hNa_v_1.4	M1476I	MYOSCN4A	DIV S5	M1638
hNa_v_1.4	A1481D	MYOSCN4A	DIV S5	A1643
hNa_v_1.4	Q1633E	MYOSCN4A	C-terminus	Q1795
hNa_v_1.4	R104H	CMS16	N-terminus	R99
hNa_v_1.4	M203K	CMS16	DI S3	F198
hNa_v_1.4	R225W	CMS16	DI S4	R220
hNa_v_1.4	S246L	CMS16	DI S5	S241
hNa_v_1.4	P382T	CMS16	DI S5-S6	P337
hNa_v_1.4	D1069N	CMS16	DIII S2	D1230
hNa_v_1.4	R1135C	CMS16	DIII S4-S5	R1299
hNa_v_1.4	C1209F	CMS16	DIII S5-S6	C1370
hNa_v_1.4	V1442E	CMS16	DIV S3-S4	V1604
hNa_v_1.4	R1454W	CMS16	DIV S4	R1616
hNa_v_1.4	R1457H	CMS16	DIV S4	R1619

PMC: Paramyotonia congenita of von Eulenburg; HOKPP2: Periodic paralysis hypokalemic 2; HYPP: Periodic paralysis hyperkalemic; NKPP: Periodic paralysis normokalemic; MYOSCN4A: Myotonia SCN4A-related; CMS16: Myasthenic syndrome, congenital, 16

**Table 6:** Structural mapping of disease-related mutations identified in human Na_v_1.5

Related proteins	Mutations	Diseases	Structural position	Map on hNa_v_1.7
hNa_v_1.5	E161K	PFHB1A	DI S2	E156
hNa_v_1.5	R225W	PFHB1A	DI S4	R220
hNa_v_1.5	G298S	PFHB1A	DI S4-S5	–
hNa_v_1.5	T512I	PFHB1A	DI - DII	V518
hNa_v_1.5	G514C	PFHB1A	DI - DII	G520
hNa_v_1.5	G752R	PFHB1A	DII S2-S3	G779
hNa_v_1.5	R1232W	PFHB1A	DIII S1-S2	K1219
hNa_v_1.5	D1275N	PFHB1A	DIII S3	D1262
hNa_v_1.5	D1595N	PFHB1A	DIII D3-S4	D1582
hNa_v_1.5	T1620K	PFHB1A	DIV S3-S4	T1607
hNa_v_1.5	G9V	LQT3	N-terminus	G8
hNa_v_1.5	R18Q	LQT3	N-terminus	K17
hNa_v_1.5	R27H	LQT3	N-terminus	R26
hNa_v_1.5	E30G	LQT3	N-terminus	E29
hNa_v_1.5	R43Q	LQT3	N-terminus	K40
hNa_v_1.5	E48K	LQT3	N-terminus	D43
hNa_v_1.5	P52S	LQT3	N-terminus	P47
hNa_v_1.5	R53Q	LQT3	N-terminus	K48
hNa_v_1.5	R104G	LQT3	N-terminus	R99
hNa_v_1.5	S115G	LQT3	N-terminus	S110
hNa_v_1.5	V125L	LQT3	N-terminus	I125
hNa_v_1.5	L212P	LQT3	DI S3-S4	L207
hNa_v_1.5	R222Q	LQT3	DI S4	R217
hNa_v_1.5	R225Q	LQT3	DI S4	R220
hNa_v_1.5	R225W	LQT3	DI S4	R220
hNa_v_1.5	V240M	LQT3	DI S5	V235
hNa_v_1.5	Q245K	LQT3	DI S5	Q240
hNa_v_1.5	V247L	LQT3	DI S5	L242
hNa_v_1.5	N275K	LQT3	DI S5-S6	N270
hNa_v_1.5	G289S	LQT3	DI S5-S6	E284
hNa_v_1.5	R340W	LQT3	DI S5-S6	T329
hNa_v_1.5	R367C	LQT3	DI S5-S6	R356
hNa_v_1.5	T370M	LQT3	DI S5-S6	T359
hNa_v_1.5	I397T	LQT3	DI S6	I386
hNa_v_1.5	L404Q	LQT3	DI S6	L393
hNa_v_1.5	N406K	LQT3	DI S6	N395
hNa_v_1.5	L409V	LQT3	DI S6	L398
hNa_v_1.5	V411M	LQT3	DI S6	V400
hNa_v_1.5	A413E	LQT3	DI S6	A402
hNa_v_1.5	A413T	LQT3	DI S6	A402
hNa_v_1.5	E462A	LQT3	DI - DII	E464
hNa_v_1.5	E462K	LQT3	DI - DII	E464
hNa_v_1.5	F530V	LQT3	DI - DII	F555
hNa_v_1.5	R535Q	LQT3	DI - DII	R562
hNa_v_1.5	R569W	LQT3	DI - DII	E596
hNa_v_1.5	S571I	LQT3	DI - DII	R598
hNa_v_1.5	A572D	LQT3	DI - DII	S599
hNa_v_1.5	A572S	LQT3	DI - DII	S599
hNa_v_1.5	A572V	LQT3	DI - DII	S599
hNa_v_1.5	Q573E	LQT3	DI - DII	S600
hNa_v_1.5	G579R	LQT3	DI - DII	S606
hNa_v_1.5	G615E	LQT3	DI - DII	N641
hNa_v_1.5	L619F	LQT3	DI - DII	L615
hNa_v_1.5	P637L	LQT3	DI - DII	–
hNa_v_1.5	G639R	LQT3	DI - DII	K666
hNa_v_1.5	P648L	LQT3	DI - DII	L675
hNa_v_1.5	E654K	LQT3	DI - DII	N681
hNa_v_1.5	L673P	LQT3	DI - DII	V700
hNa_v_1.5	R680H	LQT3	DI - DII	Q708
hNa_v_1.5	R689C	LQT3	DI - DII	R716
hNa_v_1.5	R689H	LQT3	DI - DII	R716
hNa_v_1.5	P701L	LQT3	DI - DII	P728
hNa_v_1.5	T731I	LQT3	DII S1	T758
hNa_v_1.5	Q750R	LQT3	DII S2	A777
hNa_v_1.5	D772N	LQT3	DII S2-S3	D799
hNa_v_1.5	F816Y	LQT3	DII S4	F843
hNa_v_1.5	I848F	LQT3	DII S5	I875
hNa_v_1.5	S941N	LQT3	DII - DIII	S970
hNa_v_1.5	Q960K	LQT3	DII - DIII	Q989
hNa_v_1.5	R965L	LQT3	DII - DIII	R994
hNa_v_1.5	R971C	LQT3	DII - DIII	N1000
hNa_v_1.5	C981F	LQT3	DII - DIII	–
hNa_v_1.5	A997S	LQT3	DII - DIII	E1023
hNa_v_1.5	C1004R	LQT3	DII - DIII	Y1037
hNa_v_1.5	E1053K	LQT3	DII - DIII	E1095
hNa_v_1.5	T1069M	LQT3	DII - DIII	D1111
hNa_v_1.5	A1100V	LQT3	DII - DIII	–
hNa_v_1.5	D1114N	LQT3	DII - DIII	–
hNa_v_1.5	D1166N	LQT3	DII - DIII	A1153
hNa_v_1.5	R1193Q	LQT3	DII - DIII	N1180
hNa_v_1.5	Y1199S	LQT3	DII - DIII	Y1186
hNa_v_1.5	E1225K	LQT3	DIII S1-S2	E1212
hNa_v_1.5	E1231K	LQT3	DIII S1-S2	R1218
hNa_v_1.5	F1250L	LQT3	DIII S2	F1237
hNa_v_1.5	L1283M	LQT3	DIII S3	L1270
hNa_v_1.5	E1295K	LQT3	DIII S3-S4	D1282
hNa_v_1.5	T1304M	LQT3	DIII S4	T1291
hNa_v_1.5	N1325S	LQT3	DIII S4-S5	N1312
hNa_v_1.5	A1326S	LQT3	DIII S4-S5	A1313
hNa_v_1.5	A1330P	LQT3	DIII S4-S5	A1317
hNa_v_1.5	A1330T	LQT3	DIII S4-S5	A1317
hNa_v_1.5	P1332L	LQT3	DIII S4-S5	P1319
hNa_v_1.5	S1333Y	LQT3	DIII S4-S5	S1320
hNa_v_1.5	I1334V	LQT3	DIII S4-S5	I1321
hNa_v_1.5	L1338V	LQT3	DIII S5	L1325
hNa_v_1.5	R1432S	LQT3	DIII S5-S6	V1419
hNa_v_1.5	S1458Y	LQT3	DIII S6	S1445
hNa_v_1.5	N1472S	LQT3	DIII S6	N1459
hNa_v_1.5	F1473C	LQT3	DIII S6	F1460
hNa_v_1.5	G1481E	LQT3	DIII - DIV	G1468
hNa_v_1.5	F1486L	LQT3	DIII - DIV	F1473
hNa_v_1.5	M1487L	LQT3	DIII - DIV	M1474
hNa_v_1.5	T1488R	LQT3	DIII - DIV	T1475
hNa_v_1.5	E1489D	LQT3	DIII - DIV	E1476
hNa_v_1.5	K1493R	LQT3	DIII - DIV	K1480
hNa_v_1.5	Y1495S	LQT3	DIII - DIV	Y1482
hNa_v_1.5	M1498V	LQT3	DIII - DIV	M1485
hNa_v_1.5	L1501V	LQT3	DIII - DIV	L1488
hNa_v_1.5	K1505N	LQT3	DIII - DIV	K1492
hNa_v_1.5	V1532I	LQT3	DIV S1	I1519
hNa_v_1.5	L1560F	LQT3	DIV S2	L1547
hNa_v_1.5	I1593M	LQT3	DIV S3	I1580
hNa_v_1.5	F1594S	LQT3	DIV S3	F1581
hNa_v_1.5	D1595N	LQT3	DIV S3	D1582
hNa_v_1.5	F1596I	LQT3	DIV S3	F1583
hNa_v_1.5	S1609W	LQT3	DIV S3	A1596
hNa_v_1.5	T1620K	LQT3	DIV S3-S4	T1607
hNa_v_1.5	R1623L	LQT3	DIV S4	R1610
hNa_v_1.5	R1623Q	LQT3	DIV S4	R1610
hNa_v_1.5	R1626H	LQT3	DIV S4	R1613
hNa_v_1.5	R1626P	LQT3	DIV S4	R1613
hNa_v_1.5	R1644C	LQT3	DIV S5	R1631
hNa_v_1.5	R1644H	LQT3	DIV S5	R1631
hNa_v_1.5	T1645M	LQT3	DIV S5	T1632
hNa_v_1.5	L1650F	LQT3	DIV S5	L1637
hNa_v_1.5	M1652R	LQT3	DIV S5	M1639
hNa_v_1.5	M1652T	LQT3	DIV S5	M1639
hNa_v_1.5	I1660V	LQT3	DIV S5	I1647
hNa_v_1.5	V1667I	LQT3	DIV S5	V1654
hNa_v_1.5	T1723N	LQT3	DIV S5-S6	S1710
hNa_v_1.5	R1739W	LQT3	DIV S5-S6	E1727
hNa_v_1.5	L1761F	LQT3	DIV S6	L1749
hNa_v_1.5	L1761H	LQT3	DIV S6	L1749
hNa_v_1.5	V1763M	LQT3	DIV S6	V1751
hNa_v_1.5	M1766L	LQT3	DIV S6	M1754
hNa_v_1.5	Y1767C	LQT3	DIV S6	Y1755
hNa_v_1.5	I1768V	LQT3	DIV S6	I1756
hNa_v_1.5	V1777M	LQT3	C-terminus	V1765
hNa_v_1.5	T1779M	LQT3	C-terminus	T1767
hNa_v_1.5	E1784K	LQT3	C-terminus	E1772
hNa_v_1.5	D1790G	LQT3	C-terminus	D1778
hNa_v_1.5	Y1795C	LQT3	C-terminus	Y1783
hNa_v_1.5	Y1795YD	LQT3	C-terminus	Y1783
hNa_v_1.5	D1819N	LQT3	C-terminus	A1807
hNa_v_1.5	L1825P	LQT3	C-terminus	L1813
hNa_v_1.5	R1826H	LQT3	C-terminus	L1814
hNa_v_1.5	D1839G	LQT3	C-terminus	D1827
hNa_v_1.5	R1897W	LQT3	C-terminus	K1885
hNa_v_1.5	E1901Q	LQT3	C-terminus	E1889
hNa_v_1.5	S1904L	LQT3	C-terminus	S1892
hNa_v_1.5	Q1909R	LQT3	C-terminus	Q1897
hNa_v_1.5	R1913H	LQT3	C-terminus	R1901
hNa_v_1.5	A1949S	LQT3	C-terminus	F1934
hNa_v_1.5	V1951L	LQT3	C-terminus	N1936
hNa_v_1.5	Y1977N	LQT3	C-terminus	Y1958
hNa_v_1.5	F2004L	LQT3	C-terminus	D1982
hNa_v_1.5	F2004V	LQT3	C-terminus	D1982
hNa_v_1.5	R2012C	LQT3	C-terminus	–
hNa_v_1.5	R18Q	BRGDA1	N-terminus	K17
hNa_v_1.5	R27H	BRGDA1	N-terminus	R26
hNa_v_1.5	N70K	BRGDA1	N-terminus	D65
hNa_v_1.5	D84N	BRGDA1	N-terminus	D79
hNa_v_1.5	F93S	BRGDA1	N-terminus	F88
hNa_v_1.5	I94S	BRGDA1	N-terminus	I89
hNa_v_1.5	V95I	BRGDA1	N-terminus	V90
hNa_v_1.5	R104Q	BRGDA1	N-terminus	R99
hNa_v_1.5	R104W	BRGDA1	N-terminus	R99
hNa_v_1.5	N109K	BRGDA1	N-terminus	P104
hNa_v_1.5	R121Q	BRGDA1	N-terminus	R116
hNa_v_1.5	R121W	BRGDA1	N-terminus	R116
hNa_v_1.5	K126E	BRGDA1	N-terminus	K121
hNa_v_1.5	L136P	BRGDA1	DI S1	L131
hNa_v_1.5	V146M	BRGDA1	DI S1	I141
hNa_v_1.5	E161K	BRGDA1	DI S2	E156
hNa_v_1.5	E161Q	BRGDA1	DI S2	E156
hNa_v_1.5	K175N	BRGDA1	DI S2	K170
hNa_v_1.5	A178G	BRGDA1	DI S2-S3	A173
hNa_v_1.5	C182R	BRGDA1	DI S2-S3	C177
hNa_v_1.5	A185V	BRGDA1	DI S2-S3	E180
hNa_v_1.5	T187I	BRGDA1	DI S3	T182
hNa_v_1.5	A204V	BRGDA1	DI S3	A199
hNa_v_1.5	L212Q	BRGDA1	DI S3-S4	L207
hNa_v_1.5	T220I	BRGDA1	DI S4	T215
hNa_v_1.5	R222Q	BRGDA1	DI S4	R217
hNa_v_1.5	V223L	BRGDA1	DI S4	V218
hNa_v_1.5	R225W	BRGDA1	DI S4	R220
hNa_v_1.5	A226V	BRGDA1	DI S4	A221
hNa_v_1.5	I230V	BRGDA1	DI S4	T225
hNa_v_1.5	V232I	BRGDA1	DI S4	V227
hNa_v_1.5	V240M	BRGDA1	DI S5	V235
hNa_v_1.5	Q270K	BRGDA1	DI S5	Q265
hNa_v_1.5	L276Q	BRGDA1	DI S5-S6	L271
hNa_v_1.5	H278D	BRGDA1	DI S5-S6	H273
hNa_v_1.5	R282C	BRGDA1	DI S5-S6	R277
hNa_v_1.5	R282H	BRGDA1	DI S5-S6	R277
hNa_v_1.5	V294M	BRGDA1	DI S5-S6	I289
hNa_v_1.5	V300I	BRGDA1	DI S5-S6	–
hNa_v_1.5	L315P	BRGDA1	DI S5-S6	Y304
hNa_v_1.5	G319S	BRGDA1	DI S5-S6	G308
hNa_v_1.5	T320N	BRGDA1	DI S5-S6	S319
hNa_v_1.5	L325R	BRGDA1	DI S5-S6	L314
hNa_v_1.5	P336L	BRGDA1	DI S5-S6	P325
hNa_v_1.5	G351D	BRGDA1	DI S5-S6	G340
hNa_v_1.5	G351V	BRGDA1	DI S5-S6	G340
hNa_v_1.5	T353I	BRGDA1	DI S5-S6	T342
hNa_v_1.5	D356N	BRGDA1	DI S5-S6	D345
hNa_v_1.5	R367C	BRGDA1	DI S5-S6	R356
hNa_v_1.5	R367H	BRGDA1	DI S5-S6	R356
hNa_v_1.5	R367L	BRGDA1	DI S5-S6	R356
hNa_v_1.5	M369K	BRGDA1	DI S5-S6	M358
hNa_v_1.5	W374G	BRGDA1	DI S5-S6	W363
hNa_v_1.5	R376H	BRGDA1	DI S5-S6	N365
hNa_v_1.5	G386E	BRGDA1	DI S5-S6	G375
hNa_v_1.5	G386R	BRGDA1	DI S5-S6	G375
hNa_v_1.5	V396A	BRGDA1	DI S6	V385
hNa_v_1.5	V396L	BRGDA1	DI S6	V385
hNa_v_1.5	N406S	BRGDA1	DI S6	N395
hNa_v_1.5	E439K	BRGDA1	DI - DII	D428
hNa_v_1.5	D501G	BRGDA1	DI - DII	D507
hNa_v_1.5	G514C	BRGDA1	DI - DII	G520
hNa_v_1.5	R526H	BRGDA1	DI - DII	R540
hNa_v_1.5	F532C	BRGDA1	DI - DII	A546
hNa_v_1.5	F543L	BRGDA1	DI - DII	F570
hNa_v_1.5	G552R	BRGDA1	DI - DII	G579
hNa_v_1.5	L567Q	BRGDA1	DI - DII	P594
hNa_v_1.5	G615E	BRGDA1	DI - DII	N641
hNa_v_1.5	L619F	BRGDA1	DI - DII	L615
hNa_v_1.5	R620C	BRGDA1	DI - DII	E647
hNa_v_1.5	T632M	BRGDA1	DI - DII	G659
hNa_v_1.5	P640A	BRGDA1	DI - DII	K667
hNa_v_1.5	A647D	BRGDA1	DI - DII	L674
hNa_v_1.5	P648L	BRGDA1	DI - DII	L675
hNa_v_1.5	R661W	BRGDA1	DI - DII	R688
hNa_v_1.5	H681P	BRGDA1	DI - DII	Q708
hNa_v_1.5	C683G	BRGDA1	DI - DII	C710
hNa_v_1.5	P701L	BRGDA1	DI - DII	P728
hNa_v_1.5	P717L	BRGDA1	DI - DII	P744
hNa_v_1.5	A735E	BRGDA1	DII S1-S2	A762
hNa_v_1.5	A735V	BRGDA1	DII S1-S2	A762
hNa_v_1.5	E746K	BRGDA1	DII S2	K773
hNa_v_1.5	G752R	BRGDA1	DII S2	G779
hNa_v_1.5	G758E	BRGDA1	DII S2	G785
hNa_v_1.5	M764R	BRGDA1	DII S2	M791
hNa_v_1.5	D772N	BRGDA1	DII S2-S3	D799
hNa_v_1.5	P773S	BRGDA1	DII S2-S3	P800
hNa_v_1.5	V789I	BRGDA1	DII S3	V816
hNa_v_1.5	R808P	BRGDA1	DII S4	R835
hNa_v_1.5	R814Q	BRGDA1	DII S4	R841
hNa_v_1.5	L839P	BRGDA1	DII S6	L866
hNa_v_1.5	F851L	BRGDA1	DII S6	F878
hNa_v_1.5	E867Q	BRGDA1	DII S5-S6	E894
hNa_v_1.5	R878C	BRGDA1	DII S5-S6	R907
hNa_v_1.5	R878H	BRGDA1	DII S5-S6	R907
hNa_v_1.5	H886P	BRGDA1	DII S5-S6	H915
hNa_v_1.5	F892I	BRGDA1	DII S5-S6	F921
hNa_v_1.5	R893C	BRGDA1	DII S5-S6	R922
hNa_v_1.5	R893H	BRGDA1	DII S5-S6	R922
hNa_v_1.5	C896S	BRGDA1	DII S5-S6	C925
hNa_v_1.5	E901K	BRGDA1	DII S5-S6	E930
hNa_v_1.5	S910L	BRGDA1	DII S5-S6	A939
hNa_v_1.5	C915R	BRGDA1	DII S5-S6	C944
hNa_v_1.5	L917R	BRGDA1	DII S6	I946
hNa_v_1.5	N927S	BRGDA1	DII S6	N956
hNa_v_1.5	L928P	BRGDA1	DII S6	L957
hNa_v_1.5	L935P	BRGDA1	DII S6	L964
hNa_v_1.5	R965C	BRGDA1	DII - DIII	R994
hNa_v_1.5	R965H	BRGDA1	DII - DIII	R994
hNa_v_1.5	A997T	BRGDA1	DII - DIII	Q1026
hNa_v_1.5	R1023H	BRGDA1	DII - DIII	H1050
hNa_v_1.5	E1053K	BRGDA1	DII - DIII	E1095
hNa_v_1.5	D1055G	BRGDA1	DII - DIII	D1097
hNa_v_1.5	S1079Y	BRGDA1	DII - DIII	–
hNa_v_1.5	A1113V	BRGDA1	DII - DIII	–
hNa_v_1.5	S1140T	BRGDA1	DII - DIII	S1128
hNa_v_1.5	R1193Q	BRGDA1	DII - DIII	N1180
hNa_v_1.5	S1219N	BRGDA1	DIII S1	S1206
hNa_v_1.5	E1225K	BRGDA1	DIII S1-S2	E1212
hNa_v_1.5	Y1228H	BRGDA1	DIII S1-S2	Y1215
hNa_v_1.5	R1232Q	BRGDA1	DIII S1-S2	K1219
hNa_v_1.5	R1232W	BRGDA1	DIII S1-S2	K1219
hNa_v_1.5	K1236N	BRGDA1	DIII S2	K1223
hNa_v_1.5	L1339P	BRGDA1	DIII S2	L1226
hNa_v_1.5	E1240Q	BRGDA1	DIII S2	E1227
hNa_v_1.5	D1243N	BRGDA1	DIII S2	D1230
hNa_v_1.5	V1249D	BRGDA1	DIII S2	I1236
hNa_v_1.5	E1253G	BRGDA1	DIII S2	E1240
hNa_v_1.5	G1262S	BRGDA1	DIII S2-S3	G1249
hNa_v_1.5	W1271C	BRGDA1	DIII S3	W1258
hNa_v_1.5	D1275N	BRGDA1	DIII S3	D1262
hNa_v_1.5	A1288G	BRGDA1	DIII S3-S4	A1275
hNa_v_1.5	F1293S	BRGDA1	DIII S3-S4	Y1280
hNa_v_1.5	L1311P	BRGDA1	DIII S4	L1298
hNa_v_1.5	G1319V	BRGDA1	DIII S4-S5	G1306
hNa_v_1.5	V1323G	BRGDA1	DIII S4-S5	V1310
hNa_v_1.5	P1332L	BRGDA1	DIII S4-S5	P1319
hNa_v_1.5	F1344L	BRGDA1	DIII S5	F1331
hNa_v_1.5	F1344S	BRGDA1	DIII S5	F1331
hNa_v_1.5	L1346I	BRGDA1	DIII S5	L1333
hNa_v_1.5	L1346P	BRGDA1	DIII S5	L1333
hNa_v_1.5	M1351R	BRGDA1	DIII S5	M1338
hNa_v_1.5	V1353M	BRGDA1	DIII S5	V1340
hNa_v_1.5	G1358W	BRGDA1	DIII S5-S6	G1345
hNa_v_1.5	K1359N	BRGDA1	DIII S5-S6	K1346
hNa_v_1.5	F1360C	BRGDA1	DIII S5-S6	F1347
hNa_v_1.5	C1363Y	BRGDA1	DIII S5-S6	C1350
hNa_v_1.5	S1382I	BRGDA1	DIII S5-S6	E1369
hNa_v_1.5	V1405L	BRGDA1	DIII S5-S6	V1392
hNa_v_1.5	V1405M	BRGDA1	DIII S5-S6	V1392
hNa_v_1.5	G1406E	BRGDA1	DIII S5-S6	G1393
hNa_v_1.5	G1406R	BRGDA1	DIII S5-S6	G1393
hNa_v_1.5	G1408R	BRGDA1	DIII S5-S6	G1395
hNa_v_1.5	Y1409C	BRGDA1	DIII S5-S6	Y1396
hNa_v_1.5	L1412F	BRGDA1	DIII S5-S6	L1399
hNa_v_1.5	K1419E	BRGDA1	DIII S5-S6	K1406
hNa_v_1.5	G1420R	BRGDA1	DIII S5-S6	G1407
hNa_v_1.5	A1427S	BRGDA1	DIII S5-S6	A1414
hNa_v_1.5	A1428V	BRGDA1	DIII S5-S6	A1415
hNa_v_1.5	R1432G	BRGDA1	DIII S5-S6	V1419
hNa_v_1.5	R1432S	BRGDA1	DIII S5-S6	V1419
hNa_v_1.5	G1433V	BRGDA1	DIII S5-S6	N1420
hNa_v_1.5	P1438L	BRGDA1	DIII S5-S6	P1425
hNa_v_1.5	E1441Q	BRGDA1	DIII S5-S6	E1428
hNa_v_1.5	I1448L	BRGDA1	DIII S6	I1435
hNa_v_1.5	I1448T	BRGDA1	DIII S6	I1435
hNa_v_1.5	Y1449C	BRGDA1	DIII S6	Y1436
hNa_v_1.5	V1451D	BRGDA1	DIII S6	V1438
hNa_v_1.5	N1463Y	BRGDA1	DIII S6	N1450
hNa_v_1.5	V1468F	BRGDA1	DIII S6	V1455
hNa_v_1.5	Y1494N	BRGDA1	DIII - DIV	Y1481
hNa_v_1.5	L1501V	BRGDA1	DIII - DIV	L1488
hNa_v_1.5	G1502S	BRGDA1	DIII - DIV	G1489
hNa_v_1.5	R1512W	BRGDA1	DIII - DIV	R1499
hNa_v_1.5	I1521K	BRGDA1	DIII - DIV	I1508
hNa_v_1.5	V1525M	BRGDA1	DIII - DIV	V1512
hNa_v_1.5	K1527R	BRGDA1	DIII - DIV	N1514
hNa_v_1.5	E1548K	BRGDA1	DIV S1-S2	E1535
hNa_v_1.5	A1569P	BRGDA1	DIV S2	I1556
hNa_v_1.5	F1571C	BRGDA1	DIV S2	F1558
hNa_v_1.5	E1574K	BRGDA1	DIV S2	E1561
hNa_v_1.5	L1582P	BRGDA1	DIV S2-S3	L1569
hNa_v_1.5	R1583C	BRGDA1	DIV S2-S3	R1570
hNa_v_1.5	R1583H	BRGDA1	DIV S2-S3	R1570
hNa_v_1.5	V1604M	BRGDA1	DIV S3	V1591
hNa_v_1.5	Q1613L	BRGDA1	DIV S3-S4	E1600
hNa_v_1.5	T1620M	BRGDA1	DIV S3-S4	T1607
hNa_v_1.5	R1623Q	BRGDA1	DIV S4	R1610
hNa_v_1.5	R1629Q	BRGDA1	DIV S4	R1616
hNa_v_1.5	G1642E	BRGDA1	DIV S5	G1629
hNa_v_1.5	R1644C	BRGDA1	DIV S5	R1631
hNa_v_1.5	A1649V	BRGDA1	DIV S5	A1636
hNa_v_1.5	I1660V	BRGDA1	DIV S5	I1647
hNa_v_1.5	G1661R	BRGDA1	DIV S5	G1648
hNa_v_1.5	V1667I	BRGDA1	DIV S5	V1654
hNa_v_1.5	S1672Y	BRGDA1	DIV S5	A1659
hNa_v_1.5	A1680T	BRGDA1	DIV S5-S6	A1667
hNa_v_1.5	A1698T	BRGDA1	DIV S5-S6	G1685
hNa_v_1.5	T1709M	BRGDA1	DIV S5-S6	T1696
hNa_v_1.5	T1709R	BRGDA1	DIV S5-S6	T1696
hNa_v_1.5	G1712S	BRGDA1	DIV S5-S6	G1699
hNa_v_1.5	D1714G	BRGDA1	DIV S5-S6	D1701
hNa_v_1.5	N1722D	BRGDA1	DIV S5-S6	N1709
hNa_v_1.5	C1728R	BRGDA1	DIV S5-S6	C1715
hNa_v_1.5	C1728W	BRGDA1	DIV S5-S6	C1715
hNa_v_1.5	G1740R	BRGDA1	DIV S5-S6	G1728
hNa_v_1.5	G1743E	BRGDA1	DIV S5-S6	G1731
hNa_v_1.5	G1743R	BRGDA1	DIV S5-S6	G1731
hNa_v_1.5	V1764F	BRGDA1	DIV S6	V1752
hNa_v_1.5	T1779M	BRGDA1	C-terminus	T1767
hNa_v_1.5	E1784K	BRGDA1	C-terminus	E1772
hNa_v_1.5	Y1795H	BRGDA1	C-terminus	Y1783
hNa_v_1.5	Y1795YD	BRGDA1	C-terminus	Y1783
hNa_v_1.5	Q1832E	BRGDA1	C-terminus	K1820
hNa_v_1.5	C1850S	BRGDA1	C-terminus	C1838
hNa_v_1.5	V1861I	BRGDA1	C-terminus	V1849
hNa_v_1.5	K1872N	BRGDA1	C-terminus	R1860
hNa_v_1.5	V1903L	BRGDA1	C-terminus	V1891
hNa_v_1.5	A1924T	BRGDA1	C-terminus	I1912
hNa_v_1.5	G1935S	BRGDA1	C-terminus	G1920
hNa_v_1.5	E1938K	BRGDA1	C-terminus	D1923
hNa_v_1.5	V1951L	BRGDA1	C-terminus	N1936
hNa_v_1.5	I1968S	BRGDA1	C-terminus	T1949
hNa_v_1.5	F2004L	BRGDA1	C-terminus	D1982
hNa_v_1.5	F2004V	BRGDA1	C-terminus	D1982
hNa_v_1.5	T220I	SSS1	DI S4	T215
hNa_v_1.5	A735V	SSS1	DII S1-S2	A762
hNa_v_1.5	P1298L	SSS1	DIII S3-S4	P1285
hNa_v_1.5	G1408R	SSS1	DIII S5-S6	G1395
hNa_v_1.5	D1792N	SSS1	C-terminus	E1780
hNa_v_1.5	S1710L	VF1	DIV S5-S6	S1697
hNa_v_1.5	F532C	SIDS	DI - DII	F557
hNa_v_1.5	S941N	SIDS	DII - DIII	S970
hNa_v_1.5	G1084S	SIDS	DII - DIII	–
hNa_v_1.5	S1333Y	SIDS	DIII S4-S5	S1320
hNa_v_1.5	F1705S	SIDS	DIV S5-S6	F1692
hNa_v_1.5	D1275N	ATRST1	DIII S3	D1262
hNa_v_1.5	D1275N	CMD1E	DIII S3	D1262
hNa_v_1.5	M138I	ATFB10	DI S1	M133
hNa_v_1.5	E428K	ATFB10	DI - DII	K417
hNa_v_1.5	H445D	ATFB10	DI - DII	Q434
hNa_v_1.5	N470K	ATFB10	DI - DII	S472
hNa_v_1.5	A572D	ATFB10	DI - DII	S599
hNa_v_1.5	E655K	ATFB10	DI - DII	D682
hNa_v_1.5	E1053K	ATFB10	DII - DIII	E1095
hNa_v_1.5	T1131I	ATFB10	DII - DIII	E1140
hNa_v_1.5	R1826C	ATFB10	C-terminus	L1814
hNa_v_1.5	V1951M	ATFB10	C-terminus	N1936
hNa_v_1.5	N1987K	ATFB10	C-terminus	E1967
hNa_v_1.5	R222Q	MEPPC	DI S4	R217

PFHB1A: Progressive familial heart block 1A; LQT3: Long QT syndrome 3; BRGDA1: Brugada syndrome 1; SSS1: Sick sinus syndrome 1; VF1: Familial paroxysmal ventricular fibrillation 1; SIDS: Sudden infant death syndrome; ATRST1: Atrial standstill 1; CMD1E: Cardiomyopathy, dilated 1E; ATFB10: Atrial fibrillation, familial, 10; MEPPC: Multifocal ectopic Purkinje-related premature contraction

**Table 7:** Structural mapping of disease-related mutations identified in human Na_v_1.6

Related proteins	Mutations	Diseases	Structural position	Map on hNa_v_1.7
hNa_v_1.6	D58N	EIEE13	N-terminus	D52
hNa_v_1.6	F210L	EIEE13	DI S3-S4	F204
hNa_v_1.6	G214D	EIEE13	DI S3-S4	G208
hNa_v_1.6	N215R	EIEE13	DI S3-S4	N209
hNa_v_1.6	V216D	EIEE13	DI S3-S4	V210
hNa_v_1.6	R223G	EIEE13	DI S4	R217
hNa_v_1.6	F260S	EIEE13	DI S5	F254
hNa_v_1.6	L407F	EIEE13	DI S6	L398
hNa_v_1.6	V410L	EIEE13	DI - DII	V401
hNa_v_1.6	E479V	EIEE13	DI - DII	E464
hNa_v_1.6	R530W	EIEE13	DI - DII	H515
hNa_v_1.6	R662C	EIEE13	DI - DII	Q643
hNa_v_1.6	T767I	EIEE13	DII S1	T758
hNa_v_1.6	F846S	EIEE13	DII S4	F837
hNa_v_1.6	R850Q	EIEE13	DII S4	R841
hNa_v_1.6	L875Q	EIEE13	DII S5	L866
hNa_v_1.6	A890T	EIEE13	DII S5	A881
hNa_v_1.6	V960D	EIEE13	DII S6	V951
hNa_v_1.6	N984K	EIEE13	DII - DIII	N975
hNa_v_1.6	I1327V	EIEE13	DIII S4-S5	I1321
hNa_v_1.6	L1331V	EIEE13	DIII S5	L1325
hNa_v_1.6	G1451S	EIEE13	DIII S6	G1444
hNa_v_1.6	G1451S	EIEE13	DIII S6	G1444
hNa_v_1.6	N1466K	EIEE13	DIII S6	N1459
hNa_v_1.6	N1466T	EIEE13	DIII S6	N1459
hNa_v_1.6	I1479V	EIEE13	DIII - DIV	I1472
hNa_v_1.6	E1483K	EIEE13	DIII - DIV	E1476
hNa_v_1.6	I1583T	EIEE13	DIV S2-S3	V1576
hNa_v_1.6	V1592L	EIEE13	DIV S3	V1585
hNa_v_1.6	S1596C	EIEE13	DIV S3	S1589
hNa_v_1.6	I1605R	EIEE13	DIV S3	L1598
hNa_v_1.6	R1617Q	EIEE13	DIV S4	R1610
hNa_v_1.6	L1621W	EIEE13	DIV S4	L1614
hNa_v_1.6	A1650T	EIEE13	DIV S5	A1643
hNa_v_1.6	P1719R	EIEE13	DIV S5-S6	P1713
hNa_v_1.6	N1768D	EIEE13	DIV S6	N1762
hNa_v_1.6	Q1801E	EIEE13	C-terminus	Q1795
hNa_v_1.6	E1870D	EIEE13	C-terminus	E1864
hNa_v_1.6	R1872W	EIEE13	C-terminus	R1866
hNa_v_1.6	R1872Q	EIEE13	C-terminus	R1866
hNa_v_1.6	R1872L	EIEE13	C-terminus	R1866
hNa_v_1.6	N1877S	EIEE13	C-terminus	N1871

EIEE13: Epileptic encephalopathy, early infantile, 13

**Table 8:** Structural mapping of disease-related mutations identified in human Na_v_1.8

Related proteins	Mutations	Diseases	Structural position	Map on hNa_v_1.7
hNa_v_1.8	L554P	SFN	DI - DII	–
hNa_v_1.8	M650K	SFN	DI - DII	Y729
hNa_v_1.8	A1304T	SFN	DIII S5	A1344
hNa_v_1.8	G1662S	SFN	DIV S5-S6	G1699
hNa_v_1.8	I1706V	SFN	DIV S6	I1744

SFN: Small fiber neuropathy

**Table 9:** Structural mapping of disease-related mutations identified in human Na_v_1.9

Related proteins	Mutations	Diseases	Structural position	Map on hNa_v_1.7
hNa_v_1.9	R222H	FEPS3	DI S4	R214
hNa_v_1.9	R222S	FEPS3	DI S4	R214
hNa_v_1.9	R225C	FEPS3	DI S4	R217
hNa_v_1.9	I381T	FEPS3	DI S6	V383
hNa_v_1.9	G699R	FEPS3	DII S5	G864
hNa_v_1.9	A808G	FEPS3	DII S6	A965
hNa_v_1.9	L811P	HSAN7	DII S6	L968
hNa_v_1.9	L1158P	FEPS3	DIII S4	L1301
hNa_v_1.9	V1184A	HSAN7	DIII S5	V1327

FEPS3: Episodic pain syndrome, familial, 3; HSAN7: Neuropathy, hereditary sensory and autonomic, 7

**Table 10:** Summary of sodium channelopathies

Related proteins	Diseases
hNa_v_1.1	**GEFS+2**: Generalized epilepsy with febrile seizures plus 2
	**EIEE6**: Epileptic encephalopathy, early infantile, 6
	**ICEGTC**: Intractable childhood epilepsy with generalized tonic-clonic seizures
	**FHM3**: Migraine, familial hemiplegic, 3
	**FEB3A**: Febrile seizures, familial, 3A
hNa_v_1.2	**BFIS3**: Seizures, benign familial infantile 3
	**EIEE11**: Epileptic encephalopathy, early infantile, 11
	**DS**: Dravet syndrome
hNa_v_1.3	**CPE**: Cryptogenic partial epilepsy
hNa_v_1.4	**PMC**: Paramyotonia congenita of von Eulenburg
	**HOKPP2**: Periodic paralysis hypokalemic 2
	**HYPP**: Periodic paralysis hyperkalemic
	**NKPP**: Periodic paralysis normokalemic
	**MYOSCN4A**: Myotonia SCN4A-related
	**CMS16**: Myasthenic syndrome, congenital, 16
hNa_v_1.5	**PFHB1A**: Progressive familial heart block 1A
	**LQT3**: Long QT syndrome 3
	**BRGDA1**: Brugada syndrome 1
	**SSS1**: Sick sinus syndrome 1
	**VF1**: Familial paroxysmal ventricular fibrillation 1
	**SIDS**: Sudden infant death syndrome
	**ATRST1**: Atrial standstill 1
	**CMD1E**: Cardiomyopathy, dilated 1E
	**ATFB10**: Atrial fibrillation, familial, 10
	**MEPPC**: Multifocal ectopic Purkinje-related premature contraction
hNa_v_1.6	**EIEE13**: Epileptic encephalopathy, early infantile, 13
hNa_v_1.7	**IEM**: Primary erythermalgia
	**PEPD**: Paroxysmal extreme pain disorder
	**CIP**: Indifference to pain, congenital, autosomal recessive
	**DS**: Dravet syndrome
	**SFN**: Small fiber neuropathy
	**FEB**: Febrile eizures
hNa_v_1.8	**SFN**: Small fiber neuropathy
hNa_v_1.9	**FEPS3**: Episodic pain syndrome, familial, 3
	**HSAN7**: Neuropathy, hereditary sensory and autonomic, 7

Despite significant advancement in the understanding of Na_v_ channel functions and their relevance to diseases, structural characterization of mammalian Na_v_ channels at atomic level has been challenging, partly due to the substantial technical hurdles in producing mammalian Na_v_ channel proteins in sufficient amount with acceptable purity. The two published bacterial Na_v_ channel crystal structures, Na_v_Ab (Payandeh et al., 2011) and Na_v_Rh (Zhang et al., 2012), in their full-length have greatly improved our understanding of how those channels conduct and select sodium ions on a structural basis. This is further enhanced by the recently published cryo-electron microscopy (cryo-EM) structure of the rabbit voltage-gated calcium (Ca_v_) channel Ca_v_1.1 (Wu et al., 2015; Wu et al., 2016), which, given the significant similarities between Ca_v_ and Na_v_ channels, provides an excellent base model for studying the structure and function of the mammalian Na_v_ channels in lieu of the elusive Na_v_ channel structure (Wu et al., 2015; Wu et al., 2016). In this Resource article, we have built a structure model of the human sodium channel Na_v_1.7 based on the Ca_v_1.1 cryo-EM structure (PDB code: 5GJV). Disease-related mutations of various Na_v_ channels are systematically mapped onto this Na_v_1.7 structural model. As expected, most mutations are located in the VSDs and the pore domain, which corroborate the functional disturbance associated with the various conditions. The human Na_v_1.7 structure model may also provide a useful tool for the structure-based design of drugs that are able to therapeutically target the Na_v_ channels.

## STRUCTURE MODEL OF HUMAN SODIUM CHANNEL Na_v_1.7

Homology models of the mammalian Na_v_ channels have been previously constructed based on the crystal structures of the eukaryotic potassium channels or the prokaryotic sodium channels (Tikhonov and Zhorov, 2012; Yang et al., 2012). However, the relevance of such models has been in question, since the eukaryotic sodium channels are known to be heterotetrameric while the prokaryotic sodium channels and the potassium channels are of homotetrameric nature.

We sought to build a homology-based structural model for human Na_v_1.7 because of the tremendous interest in drug development targeting this channel. The sequence identity and similarity between human Na_v_1.7 and rabbit Ca_v_1.1 are 21 and 35%, respectively (Please refer to the online Supplementary Fig. 2 of Wu et al., 2016). Importantly, the key amino acids within the VSDs and the pore domains are highly conserved (Wu et al., 2015; Wu et al., 2016). The cryo-EM structure of rabbit Ca_v_1.1 was then used as the template for homology modeling of human Na_v_1.7. The primary sequence of human Na_v_1.7 was aligned with rabbit Ca_v_1.1 in MOE with manual adjustment when necessary. The structure model of human Na_v_1.7 was created with the Homology Model module in MOE using the GB/VI scoring function with AMBER12:EHT force field (MOE, 2016).

**Figure 1:**
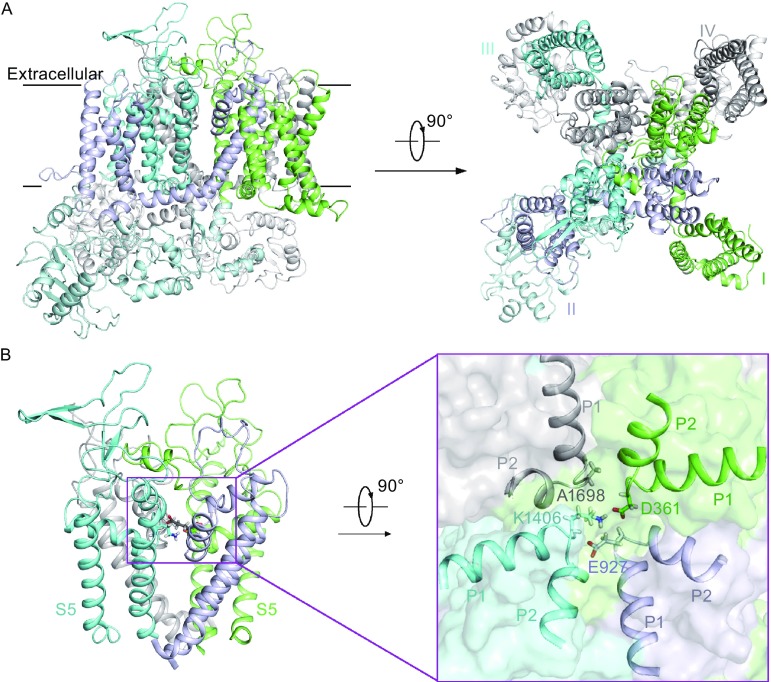
Homology model structure of human Na_v_1.7 sodium channel. (A) Intra-membrane view and extracellular view of the structure model of Na_v_1.7. The four domains are colored green, light blue, cyan, and gray for domain I, II, III, and IV, respectively. (B) The pore domain of Na_v_1.7 structure model. The S5–S6 segments of Na_v_1.7 are shown and the four selectivity filter amino acids are shown as sticks (left). A close-up view of the four SF residues, D361 in domain I, E927 in domain II, K1406 in domain III, and A1698 in domain IV (right)

The human Na_v_1.7 model structure resembles the structure of rCa_v_1.1 in general (Fig. 1A). However, the model exhibits pronounced differences from the calcium channel and bacterial sodium channels particularly in selectivity filter. The SF of Na_v_1.7 consists of four different amino acid residues DEKA (Fig. 1B). In contrast, the Ca_v_1.1 SF is constituted by four repeated essential glutamic acids, EEEE, while Na_v_Ab and Na_v_Rh contain TLESWS or TLSSWE in each protomer, respectively. This human Na_v_1.7 structure model represents the first one-chain sodium channel model with asymmetric repeats and is expected to shed new light on the mammalian sodium channel functions.

## MAPPING OF DISEASE-RELEVANT MUTATIONS ONTO THE Na_v_1.7 STRUCTURE MODEL

**Figure 2:**
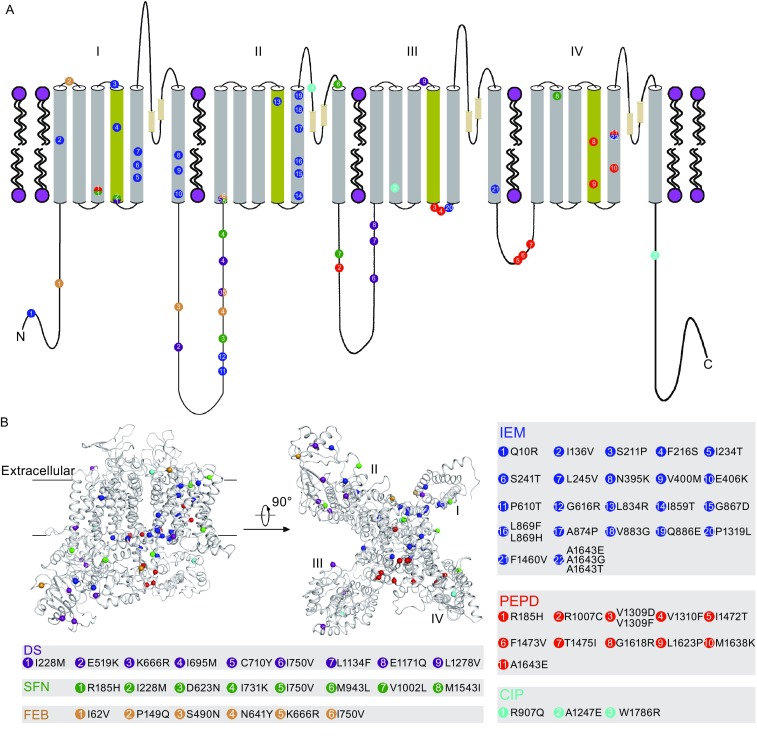
Amino acid locations of Na_v_1.7 disease-related mutations on the Na_v_1.7 structure model. (A) The topology of human Na_v_1.7 sodium channel. Cylinders represent the transmembrane segments, which are colored in gray except that the S4 voltage-sensing segments are colored in yellow. The lines represent the soluble regions between the transmembrane segments or the N/C-terminus. The two P helices between S5 and S6 segments are shown in cylinders. Mutations of Na_v_1.7 are discriminately mapped on the topology scheme of Na_v_1.7 by different colors, namely, IEM (blue), PEPD (red), CIP (cyan), DS (purple), SFN (green), and FEB (pink). (B) Intra-membrane view and intracellular views of the Na_v_1.7 structure model. Mapping of disease-related mutations onto the Na_v_1.7 structure model is highlighted by different colors. Summary of Na_v_1.7 mutations is shown in different gray boxes

Human Na_v_1.7 sodium channel is preferentially expressed in the sensory neurons of dorsal root ganglia and sympathetic ganglia neurons, particularly within the nociceptors, which is essential for perceiving pain (Djouhri et al., 2003; Dib-Hajj et al., 2013). To date, about 60 mutations of Na_v_1.7 have been found to cause human pain syndromes including IEM, PEPD, CIP, SFN (small fiber neuropathy), DS (Dravet syndrome), and FEB (febrile seizure) (Fig. 2 and Table 1). We mapped all the reported Na_v_1.7 mutations onto this Na_v_1.7 structure model (Fig. 2). Nineteen out of 22 IEM mutations are located in the highly conserved regions of VSDs and the pore domain except for the Q10R, P610T, and G616R mutations (Fig. 2). Electrophysiology study showed that IEM mutations cause a prominent shift of the activation voltage toward a more negative region or delay deactivation, which results in neuron hyperexcitability (Choi et al., 2006; Lampert et al., 2006; Choi et al., 2009; Lampert et al., 2010). For example, mutation of A1643 within the S5 segment of domain IV to glycine (A1643G) generates a significant hyperpolarizing shift (Yang et al., 2016). Our structural analysis shows that only two IEM mutations F216S and L834R are located in the S4 positively charged segment that is directly responsible for transmembrane voltage sensing and channel activation. How other IEM mutations influence voltage sensing and channel functions is yet to be elucidated.

The PEPD mutations are mostly characterized (nine out of 11) within the S4 segment, S4-S5 linker region, and the cytosolic regions of domain III and domain IV of Na_v_1.7 except for R185H and R1007C (Fig. 2A and Table 1). Specifically, I1472T, F1473V, and T1475I are within the IFMT motif (Fig. 2A), indicating that they may disturb channel inactivation. Indeed, IFMT mutations usually impair fast inactivation with consequently persistent currents (Fertleman et al., 2006). The V1309D, V1309F, and V1310F mutations are located in the S4-S5 linker region of domain III and they have been shown to cause moderate destabilization of fast inactivation (Jarecki et al., 2008). The G1618R mutation, located within the S4 segment of domain IV, impairs inactivation and retains a persistent current compared to the wild-type (WT) channel (Choi et al., 2011), while another domain IV S4 segment mutation, L1623P, significantly increases ramp current and shortens recovery time from inactivation (Suter et al., 2015). Moreover, electrophysiology study showed that M1638K mutation (within the S5 segment of domain IV) generates faster recovery from inactivation than the WT channel, producing greater currents and reducing the threshold with increased number of action potentials (Fertleman et al., 2006; Dib-Hajj et al., 2008). Another PEPD mutation, A1643E, also located in the S5 segment of domain IV, impedes channel full inactivation, which results in persistent inward currents (Estacion et al., 2008).

The CIP patients, characterized by lack of nociceptive perception, are mostly inflicted by Na_v_1.7 nonsense mutations, which result in premature protein truncations and inability to produce functional sodium channels. Only three mutations of Na_v_1.7, namely R907Q, A1247E, and W1786R, have been reported to be associated with CIP (Fig. 2 and Table 1). Diseases such as DS, SFN, and FEB are also known to be caused by Na_v_1.7 mutations (Fig. 2 and Table 1). For example, all eight SFN mutations have been characterized. Specifically, I228M, I731K, I750V, and M1543I mutations impair slow inactivation, D623N impedes slow and fast inactivation, while R185H, M943L, and V1002L mutations enhance resurgent currents (Faber et al., 2012a). On the other hand, Na_v_1.7 mutations that are associated with DS (nine mutations) and FEB (six mutations) have not been well characterized.

## MAPPING OF OTHER HUMAN SODIUM CHANNEL DISEASE-RELATED MUTATIONS ONTO THE Na_v_1.7 STRUCTURE MODEL

**Figure 3:**
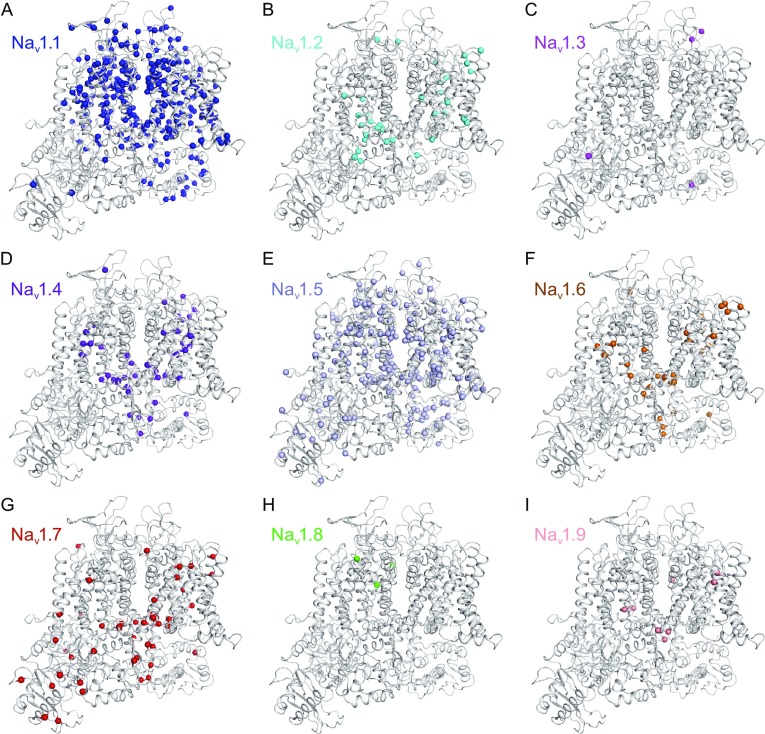
Mapping of Na_v_ channel disease-related mutations onto the Na_v_1.7 structure model. The Na_v_1.7 channel is shown in cartoon from the intra-membrane view. The Cα atoms of the disease-related amino acids are shown in spheres. Mapped mutations from nine Na_v_ sodium channels to the Na_v_1.7 structure model are differentiated by distinct colors, Na_v_1.1 (A, blue), Na_v_1.2 (B, cyan), Na_v_1.3 (C, magenta), Na_v_1.4 (D, purple blue), Na_v_1.5 (E, pale cyan), Na_v_1.6 (F, orange), Na_v_1.7 (G, red), Na_v_1.8 (H, green), and Na_v_1.9 (I, salmon)

Members of the human Na_v_ channel family share high sequence similarity and mutations of these Na_v_ channels are known to cause a vast variety of channelopathies. In order to better understand the role of those mutations in disturbing normal channel functions on a structural level, we mapped the disease-related mutations of other human Na_v_ channels onto the Na_v_1.7 structure model based on the sequence alignment reported in Wu et al., 2016 (Fig. 3).

**Figure 4:**
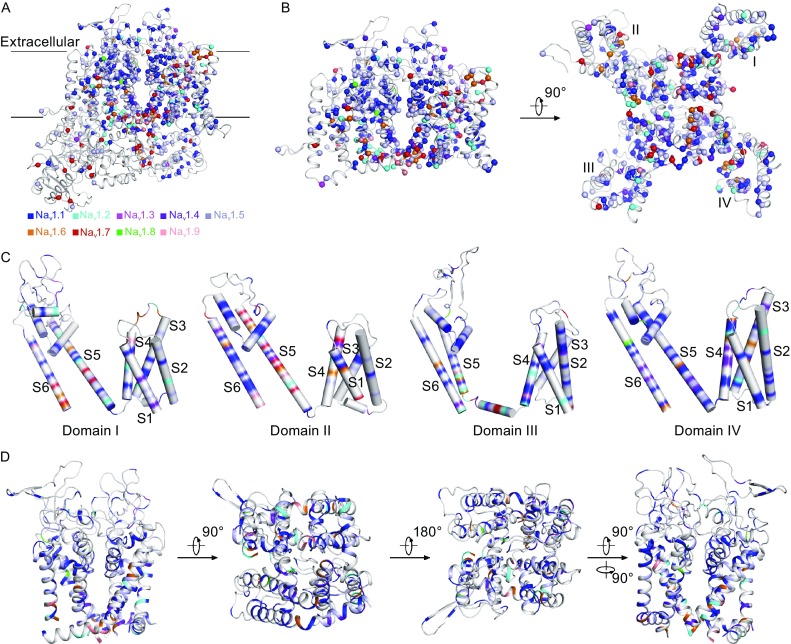
Mutations that cause sodium channelopathies are plotted on the Na_v_1.7 sodium channel model. (A) The amino acid residues related with sodium channelopathies are mapped on the Na_v_1.7 structure model. All mutated residues are shown in spheres and colored for Na_v_1.1 (blue), Na_v_1.2 (cyan), Na_v_1.3 (magenta), Na_v_1.4 (purple blue), Na_v_1.5 (pale cyan), Na_v_1.6 (orange), Na_v_1.7 (red), Na_v_1.8 (green), and Na_v_1.9 (salmon). (B) The distribution of sodium channelopathy-related mutations on the transmembrane regions of the Na_v_1.7 structure model. Mutations of the VSDs and the pore domain are shown from the intra-membrane and intracellular views. (C) The mutation distributions for the four domains. S1–S6 segments are shown in cylindrical helices. (D) Mapping mutations to the pore domain in four different views

Among all the nine Na_v_ channels, Na_v_1.1 and Na_v_1.5 have the largest numbers of reported mutations (more than 400 each) (Fig. 3A and 3E), while Na_v_1.3, Na_v_1.8, and Na_v_1.9 have the least numbers (less than 10 each) (Fig. 3C, 3H, and 3I). Notably, mutations in Na_v_1.1, Na_v_1.2, Na_v_1.3, and Na_v_1.6 mainly cause epilepsies; those in Na_v_1.4 are related to myopathies; in Na_v_1.5 result in cardiac channelopathies; and in Na_v_1.7, Na_v_1.8, and Na_v_1.9 are associated with pain-related diseases (Fig. 3 and Tables 1, 2, 3, 4, 5, 6, 7, 8, 9, 10). Mapping of all Na_v_ channel mutations onto the Na_v_1.7 structure model revealed that more than 80% of mutations are located in the VSDs and pore domains (Fig. 4A and 4B). Notably, disease-causing mutations are somewhat equally distributed in all four Na_v_ channel domains, which account for more than 20 sodium channelopathies (Fig. 4C). Furthermore, mutations are also distributed in various regions of the pore domains, suggesting that they may disturb Na_v_ channel functions by altering sodium ion selectivity and conductivity (Fig. 4D).

Na_v_1.2 mutations are largely associated with various epilepsy diseases, including BFIS3 (seizures, benign familial infantile 3), EIEE11 (epileptic encephalopathy, early infantile, 11), and DS (Fig. 3B and Table 3). More than 30 Na_v_1.2 mutations have been discovered and some of them are now functionally characterized. Interestingly, electrophysiological studies showed that Na_v_1.2 mutations can either be loss-of-function (R1319Q and L1330F) or gain-of-function (M252V, V261M, L1563V, and Y1579C) (Misra et al., 2008; Liao et al., 2010; Lauxmann et al., 2013). It is noted that BFIS3 mutations in Na_v_1.2 create less pronounced changes in the activation and inactivation potentials than the EIEE11 mutations (Shi et al., 2012).

Only six missense mutations of Na_v_1.3 have so far been identified in patients with cryptogenic partial epilepsy (Fig. 3C and Table 4). Five of them, namely K354Q, R357Q, D815N, E1160K, and M1372V, have been characterized, all of which are gain-of-function mutations, consistent with the neuronal hyperexcitability phenotype (Estacion et al., 2010; Vanoye et al., 2014).

Na_v_1.4 is essential for controlling the muscle action potential and consequently crucial for skeletal muscle contraction. Mutations of Na_v_1.4 are related with various neuromuscular disorders including PMC (paramyotonia congenita of von Eulenburg), HOKPP2 (periodic paralysis hypokalemic 2), HYPP (periodic paralysis hyperkalemic), NKPP (periodic paralysis normokalemic), MYOSCN4A (myotonia SCN4A-related), and CMS16 (myasthenic syndrome, congenital, 16) (Fig. 3D and Table 5). Different disease-causing mutations alter the Na_v_1.4 channel function through distinct mechanisms. For example, CMS16 mutations R104H, P382T, and C1209F completely abolish the Na_v_1.4 channel’s ability to conduct sodium ion, while other mutations such as M203K, R225W, and D1069N cause reduced action potential amplitude, leading to impaired channel function (Zaharieva et al., 2016). Compared to the WT channel, a CMS16 voltage sensor mutant R1457H requires longer hyperpolarization to recover which results in increased fast inactivation (Arnold et al., 2015). On the other hand, a HOKPP2 mutation R1135H (the third arginine in the domain III voltage sensor) exhibits increased depolarization, suggesting that R1135H mutation be gain-of-function (Groome et al., 2014). A MYOSCN4A mutation I582V shows a hyperpolarizing shift of 6 mV, indicating the nature of this mutation be also gain-of-function (Corrochano et al., 2014).

Na_v_1.6 is one of the sodium channels expressed in human brain and mutations of Na_v_1.6 cause EIEE13 (epileptic encephalopathy, early infantile, 13) (Fig. 3F and Table 7). More than 40 Na_v_1.6 mutations have been discovered since 2012 (Fig. 3F and Table 7), and seven of them have been studied in the functional assays. Specifically, five Na_v_1.6 mutations, namely T767I, N984K, T1716I, N1768D, and R1872W/R1872Q/R1872L, are characterized as gain-of-function, which cause hyperpolarizing shift of inactivation voltage or increased persistent current (Veeramah et al., 2012; Estacion et al., 2014; Wagnon et al., 2016), while the other two mutations, R223G and G1451S, are loss-of-function (de Kovel et al., 2014; Blanchard et al., 2015).

Five Na_v_1.8 mutations are associated with SFN, a condition that is clinically characterized by autonomic dysfunction and burning pain in the distal extremities (Fig. 3H and Table 8). Electrophysiology study has shown that Na_v_1.8 mutations, specifically L554P, A1304T, G1662S, and I1706V, accelerate inactivation recovery and enhance activation, which result in hyperexcitability (Faber et al., 2012b; Huang et al., 2013; Han et al., 2014). However, another SFN Na_v_1.8 mutation M650K causes reduced excitability of C fibers (Kist et al., 2016).

FEPS3 (episodic pain syndrome, familial, 3) and HSAN7 (neuropathy, hereditary sensory and autonomic, 7) are thought to be caused by the nine missense gain-of-function mutations of Na_v_1.9 (Fig. 3I and Table 9). Specifically, compared to the WT channel, R225C and A808G mutations induce hyperexcitability of the DRG neurons (Zhang et al., 2013), G699R enhances activation (Han et al., 2015), L811P significantly increases current density (Leipold et al., 2013), L1158P enhances spontaneous firing (Huang et al., 2014), and V1184A alters the channel voltage dependence that results in channel opening in response to hyperpolarized potentials (Leipold et al., 2015).

## DISEASE-RELATED MUTATIONS IN SODIUM CHANNELS Na_v_1.1 AND Na_v_1.5

**Figure 5:**
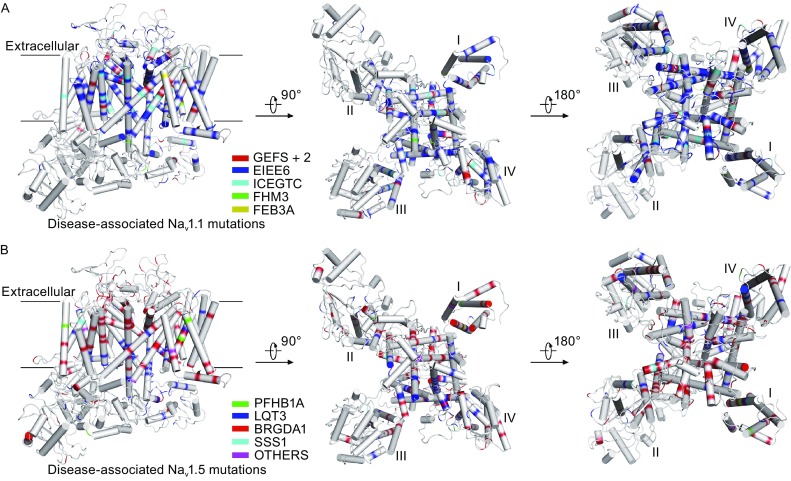
Distributions of the missense mutations in Na_v_1.1 and Na_v_1.5. (A) Distributions of Na_v_1.1 missense mutations on the Na_v_1.7 model structure. More than 400 mutations are mapped. Mutations from five Na_v_1.1-related diseases are shown from intra-membrane, intracellular, and extracellular views. The Na_v_1.7 model is shown in cylindrical helices and colored by GEFS+2 in red, EIEE6 in blue, ICEGTC in cyan, FHM3 in green, and FEB3A in yellow. (B) Distributions of Na_v_1.5 related-disease mutations on the Na_v_1.7 structure model. Mutations from Na_v_1.5 related diseases are shown from intra-membrane, intracellular, and extracellular views. Different diseases are colored in green for PFHB1A, blue for LQT3, red for BRGDA1, cyan for SSS1, and magenta for VF1, SIDS, ATRST1, CMD1E, ATFB10, and MEPPC

Mutations of Na_v_1.1 are associated with several neurological disorders including GEFS+2, EIEE6, ICEGTC, FHM3 (migraine, familial hemiplegic, 3), and FEB3A (febrile seizures, familial, 3A) (Table 2 and Table 10). More than 400 mutations of Na_v_1.1 have been identified, approximately 10% account for GEFS+2 while 80% for EIEE6 (Fig. 5A and Table 2). By mapping the Na_v_1.1-related mutations to the Na_v_1.7 structure model, we identified that most mutations are located in the VSDs and the pore domain (Fig. 5A). For example, mutations of the four positively charged residues, R1639G, R1642S, R1645Q, and R1648C, are present in the domain IV S4 segment (Table 2), suggesting that these EIEE6 mutations may alter the voltage sensing behavior of the channel. In addition, it is noteworthy that Na_v_1.1 mutations can be either loss-of-function or gain-of-function (Catterall et al., 2010; Escayg and Goldin, 2010). For example, two GEFS+2 mutations W1204R and R1648H increase the level of persistent current through gain-of-function (Lossin et al., 2002), while the loss-of-function M145T mutation in FEB3A decreases 60% of the current density (Mantegazza et al., 2005).

Na_v_1.5 is the primary sodium channel in the heart and is essential for the cardiac action potential initiation. More than 400 Na_v_1.5 mutations have been discovered and they are implicated in a wide variety of cardiac diseases—including PFHB1A (progressive familial heart block 1A), LQT3, BRGDA1, SSS1, VF1 (familial paroxysmal ventricular fibrillation 1), SIDS (sudden infant death syndrome), ATRST1 (atrial standstill 1), CMD1E (cardiomyopathy, dilated 1E), ATFB10 (atrial fibrillation, familial, 10), and MEPPC (multifocal ectopic Purkinje-related premature contractions) (Fig. 5B and Table 6). By mapping all the Na_v_1.5 mutations onto the Na_v_1.7 structure model, it shows that most mutations are located in the transmembrane regions of the channel, suggesting that these mutations might disturb voltage sensing or sodium conduction (Fig. 5B). Furthermore, about 50% of the Na_v_1.5 mutations account for BRGDA1, while 30% for LQT3. Similar to the case of Na_v_1.1, mutations in Na_v_1.5 can be either loss-of-function or gain-of-function. For example, loss-of-function mutations are associated with BRGDA1, CMD1E, SSS1, and ATFB10 (Tan et al., 2001; Smits et al., 2005; Makiyama et al., 2008; Laurent et al., 2012), while gain-of-function mutations of Na_v_1.5 are responsible for LQT3 (Remme et al., 2006), CMD1E, and ATFB10 (Olson et al., 2005), and most recently MEPPC (Swan et al., 2014).

## CONCLUDING REMARKS

The Na_v_ family of sodium channels are important drug targets for the pharmaceutical industry. However, no atomic structure of any mammalian Na_v_ channels is currently available, preventing the establishment of an in-depth structure-function relationship for this important group of sodium channels and application of structure-based approach to rationally design compounds that are able to modulate the functions of those Na_v_ channels in a disease relevant manner. Using the recently published cryo-EM structure of a rabbit Ca_v_ channel Ca_v_1.1, we established an atomic level heterotetrameric structure model for the human Na_v_ channel Na_v_1.7. Disease-related mutations of Na_v_1.7 and other members of the Na_v_ family, which are largely responsible for many neurological disorders like epilepsies, pains, and myopathies, are mapped onto the structure model. Taken together the available functional data, we attempted to establish a rudimentary structure-function relationship for human Na_v_1.7 and other members of the Na_v_ channel family. It is noticeable that sodium channelopathies can be attributed to both loss-of-function and gain-of-function mutations.

However, we must realize that the current Na_v_1.7 structural model has its limitation and the atomic resolution mammalian Na_v_ channel structure is urgently needed. In recent years, cryo-EM technology is becoming a mainstream technology for structural biology, which is able to potentially overcome the significant technical hurdles in producing challenging proteins such as mammalian Na_v_ channels in sufficient quality and the necessity of crystallization for structural elucidation. Detailed mechanisms of how the Na_v_ channels sense voltage changes and conduct sodium ions can only be answered when such atomic resolution structures become available. We hope the Na_v_1.7 structure model presented here is a temporary surrogate to help understand the Na_v_ channel functions, particularly those relevant to the various neurological diseases, at atomic level, and contributes to the structure-based rational design of the next generation Na_v_ channel modulators.

## SUMMARY OF DISEASE-RELATED MUTATIONS FOR SODIUM CHANNELS

Most of the Na_v_ channel disease-related mutations are extracted from the UNIPROT websites:

http://www.uniprot.org/uniprot/P35498 (Na_v_1.1);

http://www.uniprot.org/uniprot/Q99250 (Na_v_1.2);

http://www.uniprot.org/uniprot/P35499 (Na_v_1.4);

http://www.uniprot.org/uniprot/Q14524 (Na_v_1.5);

http://www.uniprot.org/uniprot/Q9UQD0 (Na_v_1.6);

http://www.uniprot.org/uniprot/Q15858 (Na_v_1.7);

http://www.uniprot.org/uniprot/Q9Y5Y9 (Na_v_1.8);

http://www.uniprot.org/uniprot/Q9UI33 (Na_v_1.9).

In the UNIPROT websites, there are no mutations described for Na_v_1.3. During literatures searching, we found that six mutations of Na_v_1.3 are associated with cryptogenic partial epilepsy. Except for the present mutations in the UNIPROT websites, we found additional mutations of Na_v_ channels in literatures. All mutations are summarized in Tables 1, 2, 3, 4, 5, 6, 7, 8, 9. However, we recognize that our summary may not contain all Na_v_ channel disease-related mutations owing to abundant literatures reporting Na_v_ channel disease-related mutations and increasing volume of work describing new findings.

## Electronic supplementary material

Below is the link to the electronic supplementary material.

Supplementary material 1 (PDB 2287 kb)
